# Identifying early symptoms associated with a diagnosis of childhood, adolescent and young adult cancers: a population-based nested case-control study

**DOI:** 10.1038/s41416-024-02786-5

**Published:** 2024-07-14

**Authors:** D. Saatci, J. Oke, A. Harnden, J. Hippisley-Cox

**Affiliations:** Nuffield Department of Primary Care Health Sciences, University of Oxford, Oxford, UK

**Keywords:** Cancer, Epidemiology

## Abstract

**Background:**

Childhood, teenage and young adult (CTYA, 0–24 years) cancers are rare and diverse, making timely diagnosis challenging. We aim to explore symptoms and symptom combinations associated with a subsequent cancer diagnosis and to establish their timeframe.

**Methods:**

Using the QResearch Database, we carried out a matched nested case-control study. Associations between pre-specified symptoms encountered in primary care and a subsequent diagnosis of any cancer were explored using conditional logistic regression. Median diagnostic intervals were used to split symptoms into “late” and “early” timeframes to identify relevant early symptoms.

**Results:**

3186 cases and 50,576 controls were identified from a cohort of 3,424,771 CTYA. We identified 12 novel associations, of which hemiparesis [OR 90.9 (95%CI 24.7-335.1), PPV = 1.6%], testicular swelling [OR 186.7 (95%CI 86.1-404.8), PPV = 2.4%] and organomegaly [OR 221.6 (95%CI 28.3-1735.9), PPV = 5.4%] had significant positive predictive values (PPV). Limb pain, a known marker of serious illness in children, was a recurrent early symptom across cancer subtypes. Similar clinical presentations were observed across childhood and TYA cancers.

**Discussion:**

Using the largest cohort to date, we provide novel information on the time-varying predictive utility of symptoms in the diagnosis of CTYA cancers. Our findings will help to raise clinical and public awareness of symptoms, stratify those at higher-risk and ultimately aid earlier diagnosis.

## Introduction

Cancer is one of the commonest causes of mortality-by-disease among children, teenagers and young adults (CTYA) [1] and ranks as the 6th leading cause of cancer burden worldwide [2]. Delays in cancer diagnosis may play a contributory role [3, 4]. Thus, improving opportunities for early diagnosis remains a key global priority. As screening strategies remain unfeasible for CTYA cancers [5], early diagnosis relies on the prompt recognition of symptomatic cancer. However, symptomatic cancer in this age group is challenging to detect because they are rare and often have non-specific presentations that frequently mimic self-limiting conditions [6].

In the United Kingdom (UK), where longer delays have been observed in childhood cancer diagnosis compared to other high-income countries (https://www.health.org.uk/sites/default/files/CtGtCC_HeadSmart_report.pdf) [7], one strategy to aid early detection of symptomatic CTYA cancer has been through national awareness initiatives, such as HEADSMART (https://www.health.org.uk/sites/default/files/CtGtCC_HeadSmart_report.pdf) and Child Cancer Smart [8]. HEADSMART has contributed to substantial improvements in diagnostic intervals in central nervous system (CNS) tumours [9]. However, evidence for the clinical features used in HEADSMART is largely derived from hospital-based observational studies [10], and as CTYAs with underlying cancers more commonly first present to their general practitioner (GP) in the UK [11], it is unclear if these hospital-based clinical presenting features fully represent the symptoms encountered in primary care. This is also reflected by reports that a high level of diagnostic uncertainty among GPs exists even after taking part in this initiative (https://www.health.org.uk/sites/default/files/CtGtCC_HeadSmart_report.pdf).

To date, there has been a limited number of observational studies studying the presenting features of CTYA cancers in primary care [12–14]. Symptoms identified through these studies provide a valuable starting point, however, are limited by study design (e.g., questionnaire-based) [14], absence of linkage to the National Cancer Registry, and sample size (e.g., only 8% of the population captured by primary care database Clinical Practice Research Datalink) [12, 13]. They may, therefore, be unable to capture all relevant and rarer symptoms associated with CTYA cancers. In line with this, in the UK, concerns were raised by paediatric oncologists about the reliance of national guidance on these observational studies, as it was felt that they were unable to capture all relevant clinical presentations [15]. Thus, there is still a pressing need for further observational studies to determine the symptomology of cancer in CTYA who attend primary care. Accordingly, we aimed to quantify symptoms and signs associated with a diagnosis of CTYA cancers, overall and across specific cancer subtypes, through a nested case-control study using a large representative population-based linked electronic healthcare data from the UK.

## Methods

### Data sources

QResearch Database is a nationally representative primary care database consisting of over 35 million anonymised health records from approximately 1300 general practices in England ( ~ 20% UK population) [16] (www.qresearch.org). Records consist of patient-level demographic information (i.e., year-of-birth, sex, self-assigned ethnicity), as well as clinical information, including cancer diagnoses and clinical presentations. Primary care records are linked to hospital admission, civil registration and the National Cancer Registry data, where linkage is based on an individual patient’s anonymized NHS number. This number is valid and complete in 99.8% of primary care/civil registry data and 98% of hospital admissions data (www.qresearch.org).

### Study population and design

An open cohort of children, teenagers and young adults (from birth up to 25 years) who were registered with a GP within QResearch Database between 1st January 1998 and 31st December 2018 was used to carry out a nested case-control study. Study period entry was the latest of date of registration with the practice plus 1 year, date on which the practice computer system was installed plus 1 year, and the study start date (1 January 1998). Study period exit was the earliest of cancer diagnosis, turning 25, date of death, practice exit date or study end date (31 December 2018).

### Identification of cases and controls

Cases were the commonest non-skin cancer diagnoses in this age group (https://www.cancerresearchuk.org/health-professional/cancer-statistics/) and were categorised into subtypes according to the International Classification for Childhood Cancers (third edition, ICCC-3) [17]: (1) Leukaemias and myelodysplastic diseases, (2) lymphomas and reticuloendothelial neoplasms, (3) central nervous system and intraspinal tumours, (4) soft tissue and bone sarcomas, (5) abdominal tumours (renal tumours, neuroblastomas, hepatic tumours) and (6) gonadal germ cell tumours (Supplementary Table 1). Cases were identified through recorded SNOMED-CT and International Classification of Disease (ICD-10) codes, which match the cancer classifications defined by the ICCC-3. Date of diagnosis was identified solely through the National Cancer Registry. Cases with a diagnosis of retinoblastoma were excluded. This is because (1) a substantial proportion of retinoblastomas may be captured through screening (i.e., different presentation pathways) and (2) of those that present following the neonatal period, we are unlikely to have sufficient sample size to detect associated symptoms. Similarly, “other epithelial carcinomas” were excluded as we are unlikely to have sufficient sample size to detect associated symptoms with individual subgroups within this category. As we would be unable to have sufficient power to explore each of these subgroups in detail, we made the a priori decision not to include them in our analysis. Cases with a diagnosis prior to study start date were excluded, as were those with the following pre-existing conditions linked to cancer: Down’s Syndrome, neurofibromatosis type I and II, ataxia telangiectasia, tuberous sclerosis, and Li Fraumeni [18–22].

Each case was matched to up to 20 controls, by age, sex, general practice and calendar time using incidence density sampling [23]. Incidence density sampling was used as this allows to obtain a direct estimate of the rate ratio (i.e., giving estimate of risk) and the estimates are not biased by differential loss to follow up among the exposed and unexposed [23].

### Symptom and risk factor selection

Symptom selection for each cancer type was carried out using two approaches. First, symptoms were identified through a combination of literature review [10, 24], National Institute of Health and Care Excellence (NICE) guidelines [25] and patient representative input. Second, any symptom that occurred in > 5% of either cases or controls within 6 months before diagnosis were included. SNOMED-CT codes that had similar meaning (e.g., syncope and fainting) were grouped together. Any symptoms that were not identified using the first approach was captured using the second approach. The combined approach yielded 60 symptoms overall, 29 for leukaemias, 28 for lymphomas, 27 for CNS tumours, 19 for sarcomas, 16 for abdominal tumours and 15 for gonadal germ cell tumours (Supplementary Tables 2 and 5). One symptom, eczema, not considered to be relevant to cancers in this age group was used as negative controls to explore potential recording bias.

Additionally, deprivation and ethnicity have previously been reported to be potential risk factors and were included in analyses [26, 27]. Ethnicity was defined as self- or parent-reported ethnicity on primary care health records. Ethnic groups were recorded based on the 2011 Census of England and Wales in 2 broad categories (White, Other) [28]. Level of deprivation was assessed through the Townsend deprivation score which is an area-level continuous score based on an individual’s postcode; factors that included unemployment, non-car ownership, non-home ownership, and household overcrowding, are measured for a given area of approximately 120 households, via the 2011 Census of England and Wales and combined to give a Townsend score for that area, with the first quintile representing the lowest deprivation level and the fifth quintile representing the highest deprivation level [29].

### Subgroup analysis

Subgroup analyses were carried out for each cancer subtype. Further analyses were carried out across two age groups: childhood (0–14 years) and TYA (15–24 years), to explore age-related differences in clinical presentation. As most abdominal tumours occur in children and most gonadal germ cell tumours in TYA, these two cancer types were explored only in their respective age groups (https://www.cancerresearchuk.org/health-professional/cancer-statistics/).

### Sample size calculations

Prior to carrying out analyses, sample size calculations to identify the minimum required sample size to detect odds ratios of 2 at a power of 0.8 and significance level of 0.05 for a single clinical feature for the full study sample and separately for childhood (0–14 years), TYA (15–24 years), and each cancer subtype (Supplementary Appendix Table 4). An odds ratio of 2 was selected as this was the smallest reported odds ratio in the most recent primary care-based studies exploring symptomology of CTYA cancers [12, 13]. Given the total sample size of 53,762 in this study, there is sufficient sample size to detect the selected odds ratio.

### Statistical Analysis

For descriptive analyses, continuous variables were presented as means (medians if non-normal) and categorical variables were presented in frequencies and comparisons between cancers and the general population were carried out using two-sided t-tests (Mann-Whitney U if non-normal) and χ2 tests, respectively.

The association between individual symptoms and a subsequent cancer diagnosis was explored using univariate and multivariable conditional logistic regression. A p-value threshold of 0.01 was used to select symptoms to include in multivariable analyses in order to (1) balance both clinical relevance of symptoms as well as their statistical magnitude and (2) account for multiple testing. Post-hoc analysis controlling the false discovery rate at 0.05 using the Benjamini–Hochberg method was also carried out to account for multiple testing. Symptoms that were previously reported in the most recent population-based primary care study^13,14^ were categorised as “Already Known” and any unreported symptoms were categorised as “New Associations”.

Positive predictive values were calculated using post-test odds = pre-test odds × likelihood ratio [30], where the prior odds were derived from the national incidence rates [31]. Their corresponding confidence intervals were calculated using previously established methodology [32]. Multiple imputation with chained equations was used to impute missing values for ethnicity (using multinomial regression) and Townsend Quintile (using ordered logistic regression) [29] under the missing at random assumption. In our study, ethnicity was missing for 44% and Townsend Quintile was missing for 0.4% of the total population. Thus, 50 imputed datasets were generated to account for % missingness. The imputation model was inclusive of the outcome and all candidate clinical features.

The time between the date of the symptom and the index date (i.e., the diagnostic interval) was explored for each symptom and summarised using descriptive statistics. An overall and a cancer-specific median diagnostic interval was calculated and used to split symptoms into two timeframes: “late” and “early” symptoms. Multivariable conditional logistic regression excluding the late timeframe was carried out to identify relevant early symptoms. Subsequently, each “early” symptom from the multivariable analyses was combined to another significant symptom to generate paired combinations. Odds ratios for paired combinations were calculated using conditional logistic regression.

All analyses were carried out using Stata version 17 [33] and adhere to the Strengthening the Reporting of Observational studies in Epidemiology (STROBE) [34] and Reporting of studies Conducted using Observational routinely collected health Data (RECORD) guidelines [35] (Supplementary Table 3).

## Results

From a cohort of 3,424,771 CTYA (0–24 years), we identified 3186 incident cases of cancer during the study period (1998–2018). The incidence rates for selected cancers were 164 per million person-years [95%CI 159–170 per million person-years]. Table 1 summarizes the baseline characteristics of cases and the cohort of CTYA. Compared to the cohort without cancer, cases were more commonly observed in males than females (*p* < 0.001), lower deprivation level (e.g., lower Townsend score) compared to higher deprivation levels (e.g., higher Townsend score) (*p* < 0.001) and the White ethnic group (*p* < 0.001) compared with other ethnicities.

**Table 1:** Baseline characteristics of (i) all cancers (ii) cancer subtypes and (iii) study population.

Characteristics	All cancers (*n* = 3186)	Leukaemia (*n* = 769)	Lymphoma (*n* = 672)	CNS (*n* = 617)	Sarcoma (*n* = 434)	Abdominal (*n* = 263)	Gonadal germ cell (*n* = 431)	All population (*n* = 3,424,771)
Age								
Median ± IQR (years)	5 [1,13]	7 [4,15]	17 [13,21]	11 [6,17]	15 [9,19]	4 [3,11]	21 [17,22]	13 [7,20]
Sex								
Male	1862 (58.4)	425 (55.3)	399 (59.4)	356 (57.7)	239 (55.0)	140 (53.2)	320 (74.2)	1,691,347 (49.4)
Female	1324 (41.6)	344 (44.7)	273 (40.6)	261 (42.3)	195 (45.0)	123 (46.8)	111 (25.8)	1,733,424 (50.6)
Ethnicity								
White	1407 (44.1)	338 (44.0)	283 (42.1)	277 (44.9)	190 (43.8)	136 (51.7)	161 (37.3)	1,410,215 (41.2)
Asian	269 (8.4)	77 (10.0)	74 (11.0)	40 (6.5)	27 (6.2)	11 (4.2)	39 (9.0)	284,277 (8.3)
Black	74 (2.3)	14 (1.8)	17 (2.5)	23 (3.7)	9 (2.1)	6 (2.3)	< 5	118,793 (3.4)
Other	89 (2.8)	24 (3.1)	12 (1.8)	13 (2.1)	15 (3.5)	12 (4.6)	10 (2.3)	139,568 (4.1)
Missing	1347 (42.3)	316 (41.1)	286 (42.6)	264 (42.8)	193 (44.4)	98 (37.2)	217 (50.4)	1,471,918 (43.0)
Townsend								
1 (most affluent)	778 (24.4)	179 (23.3)	174 (25.9)	167 (27.1)	105 (24.4)	60 (22.8)	92 (21.3)	691,885 (20.2)
2	704 (22.1)	186 (24.2)	133 (19.8)	135 (21.8)	86 (19.7)	63 (24.0)	93 (21.6)	666,209 (19.5)
3	618 (19.4)	153 (19.9)	134 (19.9)	113 (18.3)	87 (20.0)	46 (17.5)	85 (19.7)	672,451 (19.6)
4	562 (17.6)	117 (15.2)	120 (17.9)	108 (17.5)	69 (16.0)	52 (19.8)	90 (20.8)	698,074 (20.4)
5 (most deprived)	511 (16.1)	127 (16.6)	107 (15.9)	90 (14.6)	83 (19.0)	41 (15.6)	67 (15.6)	681,739 (19.9)
Missing	13 (0.4)	7 (0.8)	< 5	< 5	< 5	< 5	< 5	14,413 (0.4)

All data except age has been expressed in numbers (% column). Age in years is rounded to the nearest integer above.

### Symptoms associated with CTYA cancer

**Fig. 1:**
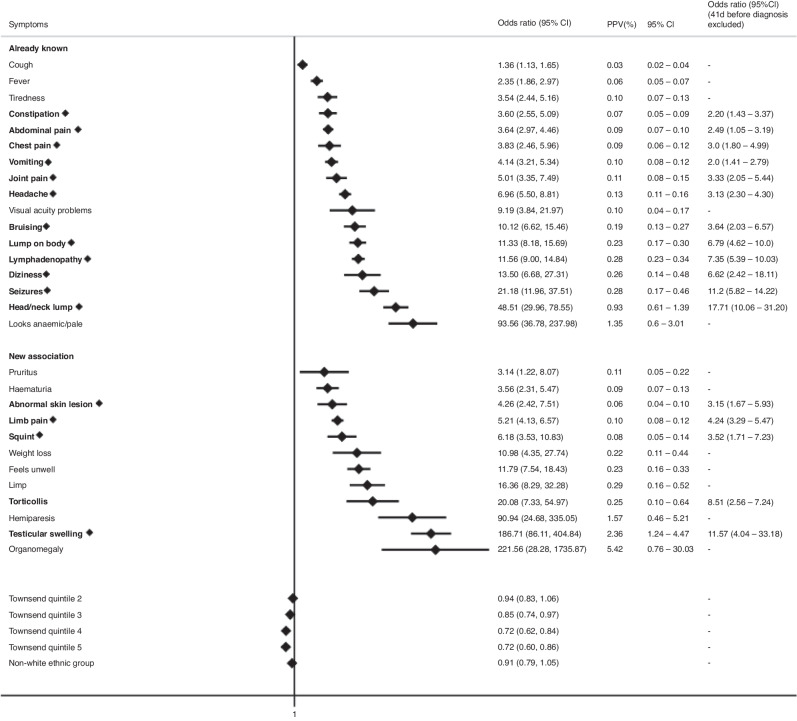
Statistically significant clinical features encountered in primary care associated with any cancer in CTYA in multivariable regression models. Early symptoms are labelled in bold with a diamond. Only statistically significant findings are reported. Already known: association previously reported in primary care, New Association: association in primary care not reported previously, OR: Odds ratio, CI: Confidence interval, PPV: Positive Predictive Value, Townsend Quintile: Deprivation level score based on individuals' area of residence (1: least deprived, 5: most deprived).

The nested case-control study had 3186 cancer cases and 50,576 controls. A total of 1644 cases (51.6%) and 9267 (18.1%) of controls had at least one of the selected symptoms recorded within 6 months prior to diagnosis. Of 39 symptoms associated with a cancer diagnosis in univariate models, 29 remained in the final multivariable model with significant *p*-value < 0.01 and FDR < 0.05 (Fig. 1). Of these, 12 symptoms have not previously been reported to be associated with cancer in a primary care setting. Additionally, abdominal mass (41/3186) was more common in cases, however, as these symptoms did not occur in the control group, odds ratios could not be calculated. Overall, there was no association between the negative control symptom (eczema) and a diagnosis of cancer in univariate analysis [OR 0.49 (95%CI 0.1–3.4), *p* = 0.5].

The median diagnostic interval for any symptom which remained in the final multivariable model was 41 days. When the last 41 days prior to diagnosis were excluded, 17 symptoms remained significant and were classified as early symptoms (Fig. 1). Of these, 6 symptoms had higher odds of being associated with cancer if in paired combination with any of the 29 relevant symptoms than if considered alone (Table 2).

**Table 2:** Strength of association between any cancer diagnosis and early symptoms (i) when considered in isolation, (ii) when considered in combination with any of the other 28 significant symptoms within 6 months of diagnosis.

Early Symptom	Isolated		In combination with any of significant symptoms	
	aOR (95% CI)	PPV (95%CI)	aOR (95%CI)	PPV (95%CI)
All age groups				
Constipation	3.60 (2.55–5.09)	0.07 (0.05–0.09)	9.54 (5.96–15.20)	0.15 (0.10–0.25)
Abdominal pain	3.64 (2.97–4.46)	0.09 (0.07–0.10)	9.15 (6.93–12.09)	0.16 (0.13–0.21)
Chest pain	3.83 (2.46–5.96)	0.09 (0.06–0.12)	12.8 (6.91–23.70)	0.25 (0.14–0.46)
Vomiting	4.14 (3.21–5.34)	0.10 (0.08–0.12)	9.52 (6.93–13.07)	0.16 (0.12–0.23)
Bruising	10.12 (6.62–15.46)	0.19 (0.13–0.27)	31.14 (16.35–59.30)	0.36 (0.13–0.99)
Limb pain	5.21 (4.13–6.57)	0.10 (0.08–0.12)	12.15 (8.62–17.12)	0.19 (0.07–0.52)
Childhood (0–14 years)				
Constipation	3.61 (2.42–5.10)	0.03 (0.02–0.05)	10.96 (6.67–17.10)	0.09 (0.06–0.16)
Abdominal pain	4.06 (3.12–5.28)	0.05 (0.04–0.06)	9.60 (6.76–13.64)	0.09 (0.06–0.12)
Vomiting	4.41 (3.31–5.88)	0.05 (0.04–0.07)	9.65 (6.73–13.86)	0.09 (0.06–0.13)
Limb pain	5.12 (3.74–7.01)	0.06 (0.04 -0.07)	14.54 (9.58–22.06)	0.09 (0.03–0.27)
TYA (15–24 years)				
Abdominal pain	3.29 (2.38–4.55)	0.02 (0.03–0.05)	8.50 (5.29–13.41)	0.08 (0.05–0.12)

*aOR* adjusted Odds Ratio, *PPV* Positive Predictive Value. Only statistically significant findings are reported.

**Fig. 2:**
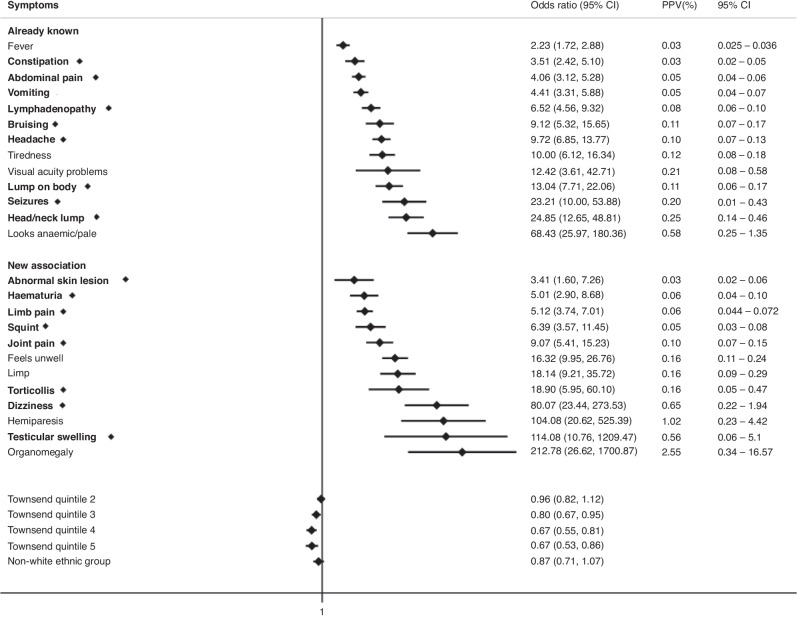
Clinical features encountered in primary care associated with any cancer in children in multivariable regression models. Early symptoms are labelled in bold with a diamond. Only statistically significant findings are reported. Already known: association previously reported in primary care, New Association: association in primary care not reported previously, OR: Odds ratio, CI: Confidence interval, PPV: Positive Predictive Value, Townsend Quintile: Deprivation level score based on individuals' area of residence (1: least deprived, 5: most deprived).

**Fig. 3:**
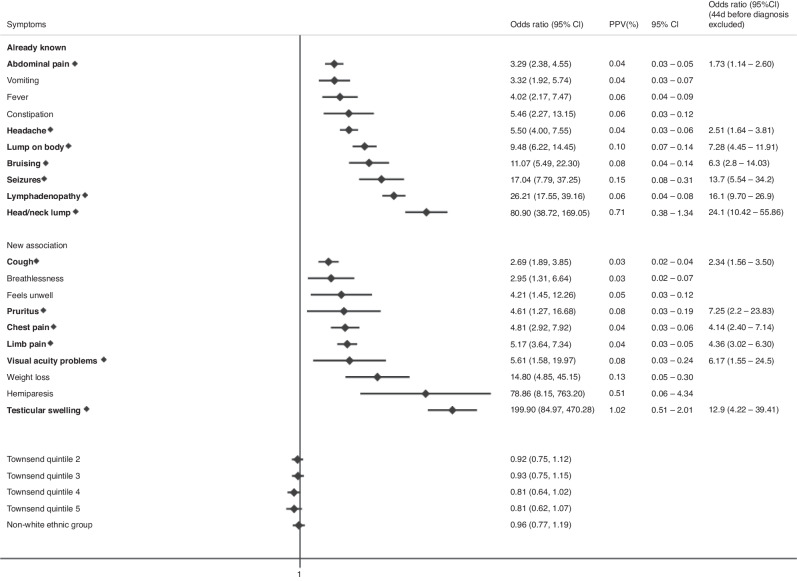
Clinical features encountered in primary care associated with any cancer in TYA in multivariable regression models. Early symptoms are labelled in bold with a diamond. Only statistically significant findings are reported. Already known: association previously reported in primary care, New Association: association in primary care not reported previously, OR: Odds ratio, CI: Confidence interval, PPV: Positive Predictive Value, Townsend Quintile: Deprivation level score based on individuals' area of residence (1: least deprived, 5: most deprived).

The final multivariable model for childhood cases (0–14 years) had 25 associated symptoms with a median diagnostic time interval of 38 days and 17 symptoms classified as “early” (Fig. 2). 4 early symptoms were associated with increased cancer risk if in paired combination than if considered alone (Table 2). For TYA cases (15–24 years), we identified 20 symptoms, and the median diagnostic interval was 44 days with 13 early symptoms, of which 1 symptom, abdominal pain, had increased odds of cancer if in paired combination (Fig. 3, Table 2). All clinical features had significant *p*-values < 0.01 and FDR < 0.05.

### Symptoms associated with specific CTYA cancers

**Fig. 4:**
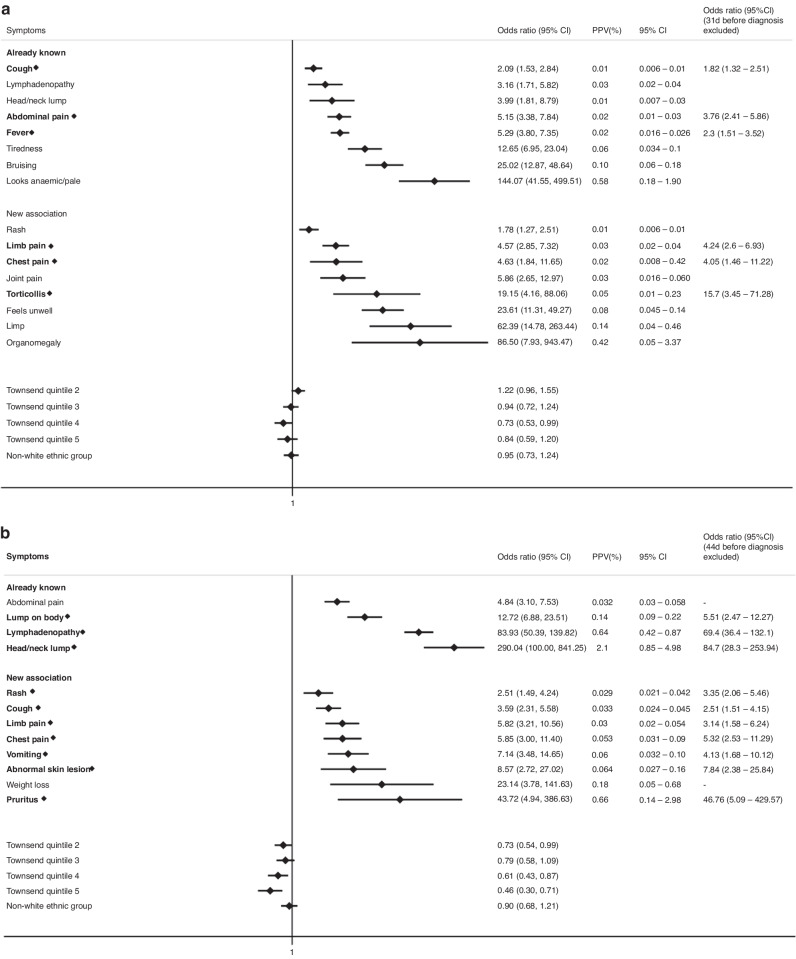

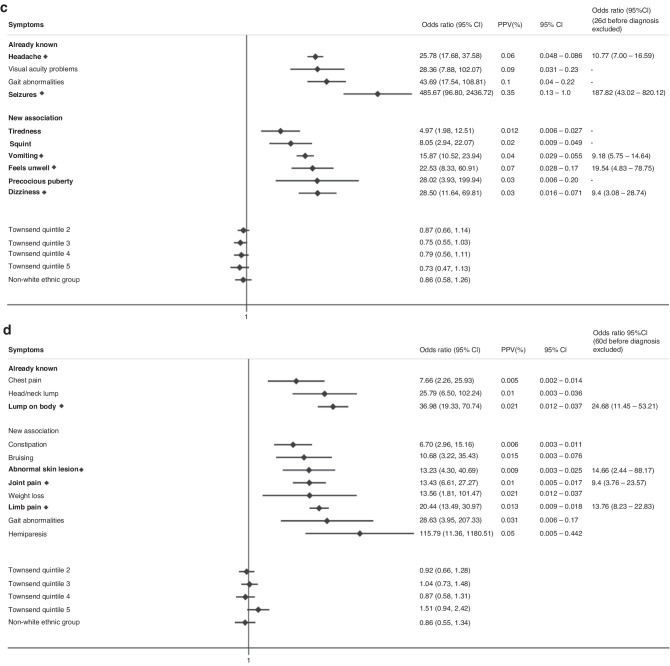

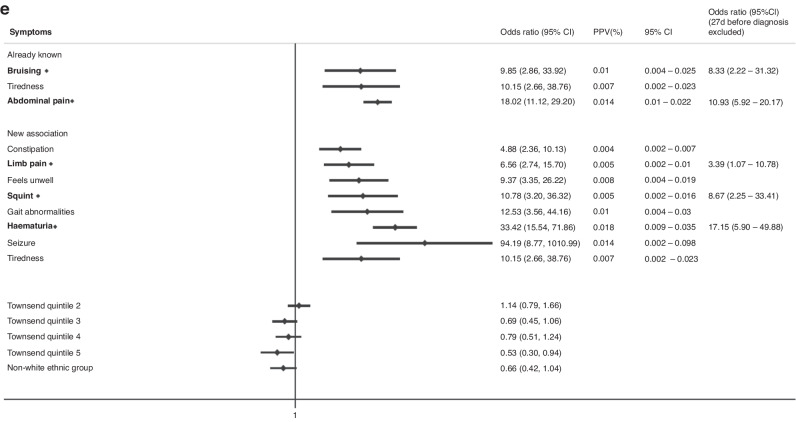
Clinical Features encountered in Primary Care associated with a CTYA cancer subtype. Then clinical features encountered in primary care associated with (**a**) leukaemia, (**b**) lymphomas, (**c**) central nervous system tumours, (**d**) bone and soft tissue sarcomas and (**e**) abdominal tumours. Early symptoms labelled in bold with a diamond. Only statistically significant findings are reported. Already known: association previously reported in primary care, New Association: association in primary care not reported previously, OR: Odds ratio, CI: Confidence interval, PPV: Positive Predictive Value, Townsend Quintile: Deprivation level score based on individuals' area of residence (1: least deprived, 5: most deprived).

We further explored symptoms associated with subsequent diagnoses of leukaemias, lymphomas, CNS tumours, sarcomas, abdominal and gonadal germ cell tumours. Supplementary Table 6 summarises the total number of cases, number of cases with symptoms and median diagnostic intervals for each cancer subtype across both age groups. Figure 4a–e and Supplementary Figures 1–4 show the odds and positive predictive values of associated symptoms (both previously reported symptoms and those identified in this study) across cancer subtypes in children and TYA, respectively.

For leukaemias, there were 19 associated symptoms in univariable models and 16 remaining in the final multivariable model with 6 “early” symptoms, which were cough, fever, torticollis, limb, chest and abdominal pain (Fig. 4a). Anaemic/pallor was most strongly associated with leukaemia diagnosis [OR 144.1 (95%CI 41.6–500)]. We explored clinically relevant interactions between (1) limb/joint pain and limp and (2) anaemia/pallor and bruising but we were unable to identify any significant interaction. When analysed across different age groups, there were 13 significant symptoms for childhood and 6 for TYA leukaemia cases (Supplementary Fig. 1). Five symptoms were identified as early symptoms in childhood leukaemias (Supplementary Fig. 1).

For lymphomas, there were 20 symptoms associated with lymphoma in univariate models, of which 12 remained in the final multivariable model and 10 “early” symptoms (Fig. 4b). Head/neck lump was most strongly associated with a lymphoma diagnosis [OR 290 (95%CI 100–841)]. We explored clinically relevant interactions between (1) lump on body and lymphadenopathy, (2) head/neck lump and lymphadenopathy, (3) chest pain and cough but we were unable to identify any significant interaction. We found 6 symptoms significant to childhood diagnoses and 12 symptoms to TYA diagnoses (Supplementary Fig. 2), of which 2 and 10 were early symptoms, respectively (Supplementary Fig. 2).

For CNS tumours, 13 symptoms were associated with a diagnosis of CNS tumours in univariate models and 10 symptoms remained in the final multivariable model (Fig. 4c). Seizures was most strongly associated with a CNS tumour diagnosis [OR 485 (95%CI 96.8 to 2436)]. Headache, seizures, vomiting, dizziness and “feels unwell” were categorised as early symptoms. We explored clinically relevant interactions between (1) dizziness and vomiting, (2) dizziness and visual acuity problems and (3) headache and visual acuity changes but were unable to find any statistically significant interaction. Both childhood and TYA CNS tumour diagnoses were associated with headache, visual acuity problems and vomiting, whilst 6 additional symptoms, gait abnormalities, seizures, squint, precocious puberty, dizziness and “feels unwell” were linked to childhood CNS tumour diagnoses (Supplementary Fig. 3). Although seizure (15/617, 2.4%) was more common in TYA cases, as it did not occur in the control group, odds ratios could not be calculated. Headache was a common early symptom for both age groups (Supplementary Fig. 3).

For sarcomas, 16 associated symptoms were identified in univariate models, with 11 remaining in the final multivariable model and 4 early symptoms, abnormal skin lesion, lump on body, limb and joint pain (Fig. 4d). Hemiparesis was most strongly associated with a sarcoma diagnosis [OR 115.8 (95%CI 11.4–1180)]. We explored clinically relevant interactions between gait abnormalities and limb/joint pain but were unable to find any statistically significant interaction. 4 symptoms (limb pain, joint pain, lump on body and abnormal skin lesions) were significantly associated with both a childhood and TYA sarcoma diagnosis (Supplementary Fig. 4).

For abdominal tumours, analyses were restricted to children (0–14 years) and 12 associated symptoms were in univariate models with 8 symptoms remaining in the final multivariable model and 5 early symptoms, bruising, abdominal pain, limb pain, squint and haematuria (Fig. 4e). We explored clinically relevant interactions between (1) gait abnormalities and limb/joint pain and (2) constipation and abdominal pain but were unable to find any statistically significant interaction.

For gonadal germ cell tumours, 3 associated symptoms were found in univariate models, with one symptom, testicular swelling remaining in the final multivariable model [OR 198.7 (95%CI 46.8–841.0)].

## Discussion

This nested case-control study of 3186 CTYA cancer cases and 50,576 controls is the largest study to date to investigate the association between clinical features encountered in primary care and a subsequent diagnosis of cancer in this age group. First, we explored these associations with all cancers grouped together to provide overall and age-specific insight into the most relevant and important symptoms that warrant further investigation. We determined clinical features which are (1) most strongly associated with a cancer diagnosis, providing support for previously reported associations as well as identifying novel associations, (2) likely to be relevant to early stages of disease onset and of these, if in combination, more likely to be associated with a cancer diagnosis. Overall, this information provides novel insight into primary care presentations of CTYA cancers and will have an important role in improving clinical and public awareness of CTYA cancers, narrowing down clinical suspicion of cancer in primary care and ultimately improving earlier diagnosis.

Overall, we identified 29 statistically significant clinical features associated with a diagnosis of CTYA cancer. Findings of this study, in addition to supporting previously reported clinical features in primary care such as pain, head/neck lumps and seizures [12, 13], provide insight into novel associations, including testicular swelling, torticollis, organomegaly, haematuria, limping and dizziness. An important novel association to highlight is the symptom coded “feels unwell”, which has previously been highlighted by young people and their parents as an important symptom that is frequently overlooked by medical professionals [36]. Furthermore, we found 17 symptoms likely to be associated with cancer at an earlier stage. Most of these symptoms were common and non-specific with low predictive values, including cough, lymphadenopathy, abdominal pain, constipation and headache. However, 3 symptoms, seizures, head/neck and testicular lumps were higher-risk symptoms, more infrequently encountered in primary care [13], which appear to be present at early-disease onset and offer a window of opportunity to be diagnosed earlier. It is possible that investigations such as imaging contribute to longer diagnostic intervals for these three symptoms and expediting investigations may speed diagnosis.

Six early symptoms had higher predictive utility when in paired combination with any of the 29 clinical features associated with a CTYA cancer diagnosis compared to when considered alone. For example, a single presentation of limb pain had an increased odds of 5.21 (95%CI 4.13–6.57), corresponding to a positive predictive value of 1 in 1000. The odds and corresponding predictive value increased to 12.2 (95%CI 8.62–17.1) and 2 in 1000, respectively, if the patient presented with limb pain and one of the other 28 significant symptoms at any other time point within 6 months. This can be useful for primary care clinicians to consider when a non-specific symptom may be associated with an underlying cancer diagnosis in this age group.

When the association between clinical features and a cancer diagnosis was explored separately across children (0–14 years) and TYA (15–24 years), 64% (16/25) of clinical features were shared across the two age groups. We found that organomegaly was only statistically significant in children, however, it was also more common in TYA (4/1473, 0.3%) and odds ratios could not be calculated as there were no patients with organomegaly in the control group. As would be expected, we also identified age-specific differences, such as squinting and limping which were only significant in children. The median diagnostic interval for TYA cancer was 6 days longer than childhood cancer [44 (IQR 15–83) vs. 38 (IQR 8–70) days, respectively]. This is in line with previous evidence and may reflect challenges around young people having their concerns acknowledged by healthcare professionals [37].

When the association between clinical features and a cancer diagnosis was explored across different types of cancers, there were distinct subtype-specific patterns in clinical presentation, which is in line with prior studies [12–14]. Leukaemias were most strongly associated with anaemia/pallor, organomegaly, bruising and limping; lymphomas with head/neck lumps as well as lymphadenopathy, and CNS tumours with seizures, visual acuity problems, headache and vomiting. Abdominal masses, haematuria and constipation were most frequently associated with abdominal tumours, whilst testicular swellings and lumps were linked to gonadal germ cell tumours. Finally, lumps and pain in extremities were commonly associated with sarcomas. There were notable differences in median diagnostic intervals, with approximately 3 weeks observed for CNS tumours and 5–8 weeks observed lymphomas and sarcomas. This is in line with previous findings that showed highly protracted diagnostic intervals for these cancers in TYA [11]. Overall, for each cancer subtype, children and TYA shared similar clinical presentations.

There were also general symptoms that recurred across several cancers and the majority were seen at earlier stages. Examples include abdominal pain and limb pain (seen in leukaemias, lymphomas, sarcomas, abdominal tumours). Limb pain, which has previously also been associated with bacterial meningitis in children, may represent an important marker of serious illness in children who present to primary care [38]. Importantly, both early symptoms had higher predictive values when considered in paired combinations. Awareness of these combinations may provide a window of opportunity for clinicians to consider an underlying cancer diagnosis at earlier stages.

Interestingly, we identified a lower overall risk of cancer for CTYA from more deprived backgrounds compared to less deprived backgrounds. Across different cancer subtypes, this trend was also observed across lymphomas, leukaemias and abdominal tumours. This is in keeping with previous epidemiological studies but should be interpreted with caution as we were unable to account for important confounders, such as maternal age and birthweight in our analyses [27].

Our study has several strengths. Firstly, it is the largest population-based study exploring the association between clinical features encountered in primary care and a subsequent diagnosis of cancer in young people. Furthermore, it is nationally representative with incidence rates in line with those reported in the UK [31]. Secondly, symptoms were selected using two approaches and took advantage of information available from existing literature, as well as from our own dataset, and provides a comprehensive list of symptoms. Thirdly, cancer cases and diagnosis dates were identified using the National Cancer Registry to ensure validity and accuracy of diagnoses [39]. Finally, our study minimises the recall, observer and selection bias associated with case-control studies through its nested design, incidence density sampling, imputation of missing data and use of routinely collected electronic healthcare records.

Our study also has several important limitations. Firstly, despite being the largest study to date, due to the rarity of CTYA cancers, we did not have sufficient power to detect symptom combinations associated with a diagnosis of specific subtypes of cancer. Furthermore, due to the rarity of these cancers, despite identifying very large odds ratios, the positive predictive values of these symptoms remain low. Secondly, we categorised cancer subtypes broadly according to the International Classification for Childhood Cancers (ICCC-3) and this may limit the interpretation of our subtype-specific results. For example, within the lymphomas category we included both Hodgkin’s and non-Hodgkin’s lymphomas, which are known to present with important differences [40] and we would not be able to capture this in our study. This is also the case with gonadal germ cell tumours, where our findings for this category was only relevant to the most common subtype in 15–24 year olds, testicular germ cell tumours, and we were unable to detect statistically significant clinical features relevant to ovarian germ cell tumours due to small numbers. Thirdly, we were unable to fully capture young people living away from their domicile (e.g., to attend university), which may affect their recorded deprivation level and thus Townsend Score. Finally, as this study is an observational study based on routinely collected data, there is the possibility of recording bias of symptoms (although we found no evidence of this bias in our study) and residual confounding from unaccounted confounders.

Overall, this is the most comprehensive study investigating symptoms associated with CTYA cancers, providing key age and cancer subtype-specific information on the predictive ability of different symptoms. Furthermore, to our knowledge this is the first study to report on symptoms which may be linked to earlier stages of the disease in this age group. We believe this information can be used to improve clinical and public awareness of the presentation of CTYA cancers and aid earlier diagnosis.

## Supplementary information

Supplementary Appendix

## Data Availability

To guarantee the confidentiality of personal and health information only the authors have had access to the data during the study in accordance with the relevant licence agreements. Access to the QResearch data is according to the information on the QResearch website (www.qresearch.org).
