# Supplementary material for: Identifying early symptoms associated with a diagnosis of childhood, adolescent and young adult cancers: a population-based nested case-control study
Source: Br J Cancer. 2024 Jul 14;131(6):1032–42. doi: 10.1038/s41416-024-02786-5 (PMC11405751; doi:10.1038/s41416-024-02786-5)

**Supplementary Appendix**

**Supplementary Table 1** Codes and flowchart used to define CTYA cancers

**Supplementary Table 2** Clinical features associated with CTYA cancer per cancer subtype

**Supplementary Table 3** STROBE Checklist

**Supplementary Table 4** Sample Size Calculations

**Supplementary Table 5** SNOMED CT definitions for symptoms/signs

**Supplementary Table 6** Number of cases with symptoms and their median diagnostic interval per cancer subtype. IQR=Inter-quartile range

**Supplementary Figure 1** Clinical features encountered in primary care associated with leukaemia in i) children, ii)TYA in multivariable regression models. Early symptoms highlighted in bold. OR= Odds ratio CI= Confidence interval PPV=Positive Predictive Value.

**Supplementary Figure 2** Clinical features encountered in primary care associated with lymphoma in i) children, ii)TYA in multivariable regression models. Early symptoms highlighted in bold. OR= Odds ratio CI= Confidence interval PPV=Positive Predictive Value.

**Supplementary Figure 3** Clinical features encountered in primary care associated with CNS tumours in i) children, ii)TYA in multivariable regression models. Early symptoms highlighted in bold. OR= Odds ratio CI= Confidence interval PPV=Positive Predictive Value.

**Supplementary Figure 4** Clinical features encountered in primary care associated with bone and soft tissue sarcomas in i) children, ii)TYA in multivariable regression models. Early symptoms highlighted in bold. OR= Odds ratio CI= Confidence interval PPV=Positive Predictive Value.

2. **Supplementary Table 1** *Codes and flowchart used to define* CTYA cancers

## *Flowchart for Cancer Classification*

## *ICD-10 codes used to define CTYA cancers*

| **Cancer** | **ICD-10 codes** |
| --- | --- |
| CNS Tumours | C70, C70.0, C70.1, C70.9, C71, C71.0, C71.1, C71.2, C71.3, C71.4, C71.5, C71.6, C71.7, C71.8, C71.9, C72, C72.0, C72.1, C72.2, C72.3, C72.4, C72.5, C72.6, C72.7, C72.8, C72.9, C79.3, C79.4 |
| Lymphoma | C81,C81.0, C81.1, C81.2, C81.3, C81.4, C81.7,C81.9, C82, C82.0, C82.1, C82.2, C82.3, C82.4, C82.5, C82.6, C82.7,C82.9 C83, C83.0, C83.1, C83.3, C83.5, C83.7,C83.9, C84, C84.0, C84.1, C84.44, C81.3, C81.4, C81.7,C81.9 C85, C85.1, C85.2, C85.7, C85.9 |
| Leukaemia | C91, C91.0, C91.1, C91.3, C91.4, C91.5, C91.6, C91.7, C91.8, C91.9,  C92, C92.0, C92.1, C92.2, C92.3, C92.4, C92.5, C92.6, C92.7, C92.8, C92.9  C93, C93.0, C93.1, C93.3, C93.7, C93.9  C94, C94.0, C94.2, C94.3, C94.4, C94.6, C94.7  C95, C95.0, C95.1, C95.7, C95.9 |
| Sarcoma | C40, C40.0, C40.1, C40.2, C40.3, C40.8, C40.9  C41, C41.0, C41.1, C41.2, C41.3, C41.4, C41.8, C41.9  C46, C46.1, C46.2, C46.3, C46.7, C46.8, C46.9  C49, C49.0, C49.1, C49.2, C49.3, C49.4, C49.5, C49.6, C49.8 C49.9 |
| Abdominal Tumours | C64, C64.X, C65, C65.X, C74. 9, C22. 2 |
| Gonadal germ cell tumours | C56, C62 |

***1.3 SNOMED-CT Definitions for CTYA Cancers***

| **Leukemias, myeloproliferative disease and myelodysplastic diseases** |
| --- |
| Acute myeloid leukemia, no International Classification of Diseases for Oncology subtype (morphologic abnormality) |
| Acute promyelocytic leukemia with PML::RARA fusion (morphologic abnormality) |
| Acute myelomonocytic leukemia (morphologic abnormality) |
| Myeloid sarcoma (morphologic abnormality) |
| Myeloid leukemia, no International Classification of Diseases for Oncology subtype (morphologic abnormality) |
| Chronic myelogenous leukemia, no International Classification of Diseases for Oncology subtype (morphologic abnormality) |
| Acute lymphoid leukemia, disease (disorder) |
| Acute myeloid leukemia (disorder) |
| Chronic lymphoid leukemia, disease (disorder) |
| Chronic myeloid leukemia (disorder) |
| Myeloid sarcoma, disease (disorder) |
| Myelodysplastic syndrome (disorder) |
| Acute promyelocytic leukemia, FAB M3 (disorder) |
| Acute myelomonocytic leukemia, FAB M4 (disorder) |
| Prolymphocytic leukemia (disorder) |
| Adult T-cell leukemia/lymphoma (disorder) |
| Chronic myelomonocytic leukemia (disorder) |
| Myelodysplastic neoplasm (morphologic abnormality) |
| Chronic myelomonocytic leukemia (morphologic abnormality) |
| Juvenile myelomonocytic leukemia (morphologic abnormality) |
| Leukemia - category (morphologic abnormality) |
| Myeloid leukemia - category (morphologic abnormality) |
| Lymphoid leukemia (disorder) |
| Subacute lymphoid leukemia (disorder) |
| Aleukemic lymphoid leukemia (disorder) |
| Adult T-cell leukemia (disorder) |
| Myeloid leukemia (disorder) |
| Chronic eosinophilic leukemia (disorder) |
| Chronic neutrophilic leukemia (disorder) |
| Subacute myeloid leukemia (disorder) |
| Chloroma (disorder) |
| Granulocytic sarcoma (disorder) |
| Aleukemic myeloid leukemia (disorder) |
| Myelomonocytic leukemia (disorder) |
| Subacute myelomonocytic leukemia (disorder) |
| Angioendotheliomatosis (disorder) |
| B-cell chronic lymphocytic leukemia (disorder) |
| T-cell prolymphocytic leukemia (disorder) |
| B-cell acute lymphoblastic leukemia (disorder) |
| Myelodysplastic syndrome with isolated del(5q) (disorder) |
| B-cell prolymphocytic leukemia (disorder) |
| Juvenile myelomonocytic leukemia (disorder) |
| Acute myeloid leukemia with myelodysplasia-related changes (disorder) |
| Myelodysplastic/myeloproliferative disease (disorder) |
| Philadelphia chromosome positive chronic myelogenous leukemia (disorder) |
| Philadelphia chromosome negative chronic myelogenous leukemia (disorder) |
| Acute myeloid leukaemia with 11q23 abnormality (disorder) |
| Clinical stage A chronic lymphocytic leukaemia (disorder) |
| Clinical stage B chronic lymphocytic leukaemia (disorder) |
| Clinical stage C chronic lymphocytic leukaemia (disorder) |

| **Lymphomas and Reticuloendothelial neoplasms** |
| --- |
| Non-Hodgkin lymphoma, no International Classification of Diseases for Oncology subtype (morphologic abnormality) |
| Hodgkin lymphoma, no International Classification of Diseases for Oncology subtype (morphologic abnormality) |
| Hodgkin lymphoma, lymphocyte depletion, diffuse fibrosis (morphologic abnormality) |
| Hodgkin lymphoma, nodular sclerosis, cellular phase (morphologic abnormality) |
| Hodgkin lymphoma, mixed cellularity (morphologic abnormality) |
| Hodgkin lymphoma, nodular sclerosis, grade 2 (morphologic abnormality) |
| Hodgkin lymphoma, nodular sclerosis, grade 1 (morphologic abnormality) |
| Hodgkin sarcoma [obs] (morphologic abnormality) |
| Hodgkin lymphoma, nodular sclerosis (morphologic abnormality) |
| Langerhans cell histiocytosis (disorder) |
| Hodgkin lymphoma, nodular lymphocyte predominance (morphologic abnormality) |
| Hodgkin lymphoma, lymphocyte depletion, reticular (morphologic abnormality) |
| Hodgkin granuloma [obs] (morphologic abnormality) |
| Burkitt lymphoma (morphologic abnormality) |
| Mastocytoma (morphologic abnormality) |
| Letterer-Siwe disease of intra-abdominal lymph nodes (disorder) |
| Letterer-Siwe disease of intrapelvic lymph nodes (disorder) |
| Letterer-Siwe disease of intrathoracic lymph nodes (disorder) |
| Letterer-Siwe disease of lymph nodes of axilla AND/OR upper limb (disorder) |
| Letterer-Siwe disease of lymph nodes of head, face AND/OR neck (disorder) |
| Letterer-Siwe disease of lymph nodes of inguinal region AND/OR lower limb (disorder) |
| Letterer-Siwe disease of lymph nodes of multiple sites (disorder) |
| Letterer-Siwe disease of spleen (disorder) |
| Leukemic reticuloendotheliosis of intra-abdominal lymph nodes (disorder) |
| Leukemic reticuloendotheliosis of intrapelvic lymph nodes (disorder) |
| Leukemic reticuloendotheliosis of intrathoracic lymph nodes (disorder) |
| Leukemic reticuloendotheliosis of lymph nodes of multiple sites (disorder) |
| Malignant histiocytosis of intra-abdominal lymph nodes (disorder) |
| Malignant histiocytosis of intrapelvic lymph nodes (disorder) |
| Malignant histiocytosis of intrathoracic lymph nodes (disorder) |
| Malignant histiocytosis of lymph nodes of multiple sites (disorder) |
| Malignant histiocytosis of spleen (disorder) |
| Malignant lymphoma of intra-abdominal lymph nodes (disorder) |
| Malignant lymphoma of intrapelvic lymph nodes (disorder) |
| Malignant lymphoma of intrathoracic lymph nodes (disorder) |
| Malignant lymphoma of lymph nodes of axilla AND/OR upper limb (disorder) |
| Malignant lymphoma of lymph nodes of head, face AND/OR neck (disorder) |
| Malignant lymphoma of lymph nodes of inguinal region AND/OR lower limb (disorder) |
| Malignant lymphoma of lymph nodes of multiple sites (disorder) |
| Malignant lymphoma of spleen (disorder) |
| Hodgkin's disease, lymphocytic-histiocytic predominance of intra-abdominal lymph nodes (disorder) |
| Hodgkin's disease, lymphocytic-histiocytic predominance of intrapelvic lymph nodes (disorder) |
| Hodgkin's disease, lymphocytic-histiocytic predominance of intrathoracic lymph nodes (disorder) |
| Hodgkin's disease, lymphocytic-histiocytic predominance of spleen (disorder) |
| Hodgkin's disease, mixed cellularity of spleen (disorder) |
| Hodgkin's disease, nodular sclerosis of spleen (disorder) |
| Hodgkin's disease of intra-abdominal lymph nodes (disorder) |
| Hodgkin's disease of intrapelvic lymph nodes (disorder) |
| Hodgkin's disease of intrathoracic lymph nodes (disorder) |
| Hodgkin's disease of lymph nodes of axilla AND/OR upper limb (disorder) |
| Hodgkin's disease of lymph nodes of head, face AND/OR neck (disorder) |
| Hodgkin's disease of lymph nodes of inguinal region AND/OR lower limb (disorder) |
| Hodgkin's disease of lymph nodes of multiple sites (disorder) |
| Hodgkin's disease of spleen (disorder) |
| Hodgkin's granuloma of intrapelvic lymph nodes (disorder) |
| Hodgkin's granuloma of intrathoracic lymph nodes (disorder) |
| Hodgkin's granuloma of spleen (disorder) |
| Hodgkin's paragranuloma of lymph nodes of axilla AND/OR upper limb (disorder) |
| Hodgkin's paragranuloma of lymph nodes of head, face AND/OR neck (disorder) |
| Hodgkin's paragranuloma of lymph nodes of inguinal region AND/OR lower limb (disorder) |
| Hodgkin's paragranuloma of spleen (disorder) |
| Hodgkin's sarcoma of intra-abdominal lymph nodes (disorder) |
| Hodgkin's sarcoma of intrapelvic lymph nodes (disorder) |
| Hodgkin's sarcoma of intrathoracic lymph nodes (disorder) |
| Hodgkin's sarcoma of spleen (disorder) |
| Mycosis fungoides of intra-abdominal lymph nodes (disorder) |
| Mycosis fungoides of intrapelvic lymph nodes (disorder) |
| Mycosis fungoides of intrathoracic lymph nodes (disorder) |
| Mycosis fungoides of lymph nodes of axilla AND/OR upper limb (disorder) |
| Mycosis fungoides of lymph nodes of head, face AND/OR neck (disorder) |
| Mycosis fungoides of lymph nodes of inguinal region AND/OR lower limb (disorder) |
| Mycosis fungoides of spleen (disorder) |
| Nodular lymphoma of intra-abdominal lymph nodes (disorder) |
| Nodular lymphoma of intrapelvic lymph nodes (disorder) |
| Nodular lymphoma of intrathoracic lymph nodes (disorder) |
| Nodular lymphoma of lymph nodes of multiple sites (disorder) |
| Nodular lymphoma of spleen (disorder) |
| Sézary's disease of spleen (disorder) |
| Diffuse non-Hodgkin's lymphoma (disorder) |
| Diffuse non-Hodgkin's lymphoma, lymphoblastic (disorder) |
| Diffuse non-Hodgkin's lymphoma, immunoblastic (disorder) |
| Diffuse non-Hodgkin's lymphoma, large cell (disorder) |
| T-zone lymphoma (disorder) |
| Lymphoepithelioid lymphoma (disorder) |
| Peripheral T-cell lymphoma (disorder) |
| T-cell lymphoma (disorder) |
| Histiocytic sarcoma (disorder) |
| Hodgkin lymphoma, lymphocyte depletion (morphologic abnormality) |
| Hodgkin's disease (disorder) |
| Malignant lymphoma (disorder) |
| Non-Hodgkin's lymphoma (disorder) |
| Hodgkin's granuloma (disorder) |
| Hodgkin lymphoma, nodular lymphocyte predominance (disorder) |
| Hodgkin's sarcoma (disorder) |
| Hodgkin lymphoma, lymphocyte-rich (disorder) |
| Hodgkin's disease, nodular sclerosis (disorder) |
| Hodgkin's disease, mixed cellularity (disorder) |
| Hodgkin's disease, lymphocytic depletion (disorder) |
| Sézary's disease (disorder) |
| Malignant histiocytosis (disorder) |
| Langerhans cell histiocytosis, disseminated (disorder) |
| Malignant mast cell tumor (disorder) |
| Burkitt's lymphoma (disorder) |
| Mycosis fungoides (disorder) |
| Hodgkin lymphoma, lymphocyte-rich (morphologic abnormality) |
| Primary cutaneous CD30 antigen positive large T-cell lymphoma (disorder) |
| Hodgkin lymphoma - category (morphologic abnormality) |
| Eosinophilic granuloma (disorder) |
| Burkitt's lymphoma of lymph nodes of head, face and neck (disorder) |
| Burkitt's lymphoma of intrathoracic lymph nodes (disorder) |
| Burkitt's lymphoma of intra-abdominal lymph nodes (disorder) |
| Burkitt lymphoma of lymph nodes of axilla and upper limb (disorder) |
| Burkitt lymphoma of lymph nodes of inguinal region and lower limb (disorder) |
| Burkitt's lymphoma of intrapelvic lymph nodes (disorder) |
| Burkitt's lymphoma of spleen (disorder) |
| Burkitt's lymphoma of lymph nodes of multiple sites (disorder) |
| Hodgkin's paragranuloma of intrathoracic lymph nodes (disorder) |
| Hodgkin's paragranuloma of intrapelvic lymph nodes (disorder) |
| Hodgkin's paragranuloma of lymph nodes of multiple sites (disorder) |
| Hodgkin's granuloma of lymph nodes of head, face and neck (disorder) |
| Hodgkin's granuloma of intra-abdominal lymph nodes (disorder) |
| Hodgkin's granuloma of lymph nodes of axilla and upper limb (disorder) |
| Hodgkin's granuloma of lymph nodes of inguinal region and lower limb (disorder) |
| Hodgkin's granuloma of lymph nodes of multiple sites (disorder) |
| Hodgkin's sarcoma of lymph nodes of head, face and neck (disorder) |
| Hodgkin's sarcoma of lymph nodes of axilla and upper limb (disorder) |
| Hodgkin's sarcoma of lymph nodes of inguinal region and lower limb (disorder) |
| Hodgkin's sarcoma of lymph nodes of multiple sites (disorder) |
| Hodgkin's disease, lymphocytic-histiocytic predominance of lymph nodes of head, face and neck (disorder) |
| Hodgkin's disease, lymphocytic-histiocytic predominance of lymph nodes of axilla and upper limb (disorder) |
| Hodgkin's disease, lymphocytic-histiocytic predominance of lymph nodes of inguinal region and lower limb (disorder) |
| Hodgkin's disease, lymphocytic-histiocytic predominance of lymph nodes of multiple sites (disorder) |
| Hodgkin's disease, nodular sclerosis of lymph nodes of head, face and neck (disorder) |
| Hodgkin's disease, nodular sclerosis of intrathoracic lymph nodes (disorder) |
| Hodgkin's disease, nodular sclerosis of intra-abdominal lymph nodes (disorder) |
| Hodgkin's disease, nodular sclerosis of lymph nodes of axilla and upper limb (disorder) |
| Hodgkin's disease, nodular sclerosis of lymph nodes of inguinal region and lower limb (disorder) |
| Hodgkin's disease, nodular sclerosis of intrapelvic lymph nodes (disorder) |
| Hodgkin's disease, nodular sclerosis of lymph nodes of multiple sites (disorder) |
| Hodgkin's disease, mixed cellularity of lymph nodes of head, face and neck (disorder) |
| Hodgkin's disease, mixed cellularity of intrathoracic lymph nodes (disorder) |
| Hodgkin's disease, mixed cellularity of intra-abdominal lymph nodes (disorder) |
| Hodgkin's disease, mixed cellularity of lymph nodes of axilla and upper limb (disorder) |
| Hodgkin's disease, mixed cellularity of lymph nodes of inguinal region and lower limb (disorder) |
| Hodgkin's disease, mixed cellularity of intrapelvic lymph nodes (disorder) |
| Hodgkin's disease, mixed cellularity of lymph nodes of multiple sites (disorder) |
| Hodgkin's disease, lymphocytic depletion of lymph nodes of head, face and neck (disorder) |
| Hodgkin's disease, lymphocytic depletion of intrathoracic lymph nodes (disorder) |
| Hodgkin's disease, lymphocytic depletion of intra-abdominal lymph nodes (disorder) |
| Hodgkin's disease, lymphocytic depletion of lymph nodes of axilla and upper limb (disorder) |
| Hodgkin's disease, lymphocytic depletion of lymph nodes of inguinal region and lower limb (disorder) |
| Hodgkin's disease, lymphocytic depletion of intrapelvic lymph nodes (disorder) |
| Hodgkin's disease, lymphocytic depletion of spleen (disorder) |
| Hodgkin's disease, lymphocytic depletion of lymph nodes of multiple sites (disorder) |
| Nodular lymphoma of lymph nodes of head, face and neck (disorder) |
| Nodular lymphoma of lymph nodes of axilla and upper limb (disorder) |
| Nodular lymphoma of lymph nodes of inguinal region and lower limb (disorder) |
| Mycosis fungoides of lymph nodes of multiple sites (disorder) |
| Sézary's disease of lymph nodes of head, face and neck (disorder) |
| Sézary's disease of intrathoracic lymph nodes (disorder) |
| Sézary's disease of intra-abdominal lymph nodes (disorder) |
| Sézary's disease of lymph nodes of axilla and upper limb (disorder) |
| Sézary's disease of lymph nodes of inguinal region and lower limb (disorder) |
| Sézary's disease of intrapelvic lymph nodes (disorder) |
| Sézary's disease of lymph nodes of multiple sites (disorder) |
| Malignant histiocytosis of lymph nodes of head, face and neck (disorder) |
| Malignant histiocytosis of lymph nodes of axilla and upper limb (disorder) |
| Malignant histiocytosis of lymph nodes of inguinal region and lower limb (disorder) |
| Leukemic reticuloendotheliosis of lymph nodes of head, face and neck (disorder) |
| Leukemic reticuloendotheliosis of lymph nodes of axilla and upper limb (disorder) |
| Leukemic reticuloendotheliosis of lymph nodes of inguinal region and lower limb (disorder) |
| Malignant mast cell tumors (disorder) |
| Mast cell malignancy of lymph nodes of head and neck (disorder) |
| Mast cell malignancy of intrathoracic lymph nodes (disorder) |
| Mast cell malignancy of intra-abdominal lymph nodes (disorder) |
| Mast cell malignancy of lymph nodes of axilla and upper limb (disorder) |
| Mast cell malignancy of lymph nodes of inguinal region and lower limb (disorder) |
| Mast cell malignancy of intrapelvic lymph nodes (disorder) |
| Malignant mast cell sarcoma of spleen (disorder) |
| Mast cell malignancy of lymph nodes of multiple sites (disorder) |
| Follicular non-Hodgkin's mixed small cleaved and large cell lymphoma (disorder) |
| Diffuse malignant lymphoma - small non-cleaved cell (disorder) |
| Malignant lymphoma - small cleaved cell (disorder) |
| Malignant lymphoma - mixed small and large cell (disorder) |
| Diffuse non-Hodgkin's lymphoma undifferentiated (diffuse) (disorder) |
| Malignant immunoproliferative small intestinal disease (disorder) |
| Malignant tumor of lymphoid, hemopoietic AND/OR related tissue (disorder) |
| Nodular lymphoma (disorder) |
| Immunoproliferative neoplasm (disorder) |
| Lymphoreticular tumor (disorder) |
| Cutaneous/peripheral T-cell lymphoma (disorder) |
| Low grade B-cell lymphoma (disorder) |
| Mucosa-associated lymphoma (disorder) |
| Follicular malignant lymphoma - small cleaved cell (disorder) |
| Follicular malignant lymphoma - large cell (disorder) |
| Enteropathy-associated T-cell lymphoma (disorder) |
| Diffuse malignant lymphoma - centroblastic (disorder) |
| Follicular non-Hodgkin's lymphoma (disorder) |
| Mast cell malignancy (disorder) |
| Primary cutaneous T-cell lymphoma (disorder) |
| Subcutaneous panniculitic cutaneous T-cell lymphoma (disorder) |
| Extranodal natural killer/T-cell lymphoma, nasal type (disorder) |
| Mantle cell lymphoma (disorder) |
| Primary mediastinal (thymic) large B-cell lymphoma (disorder) |
| Blastic plasmacytoid dendritic cell neoplasm (disorder) |
| Extranodal marginal zone B-cell lymphoma of mucosa-associated lymphoid tissue (disorder) |
| Hepatosplenic T-cell lymphoma (disorder) |
| Sarcoma of dendritic cells (accessory cells) (disorder) |
| Anaplastic lymphoma kinase negative anaplastic large cell lymphoma (disorder) |
| Diffuse follicle center lymphoma (disorder) |
| Anaplastic lymphoma kinase positive anaplastic large cell lymphoma (disorder) |
| Other malignant neoplasm of lymphoid and histiocytic tissue (disorder) |
| Follicular lymphoma grade 1 (disorder) |
| Follicular lymphoma grade 2 (disorder) |
| Follicular lymphoma grade 3 (disorder) |
| Follicular lymphoma grade 3a (disorder) |
| Follicular lymphoma grade 3b (disorder) |
| Diffuse large B-cell lymphoma (disorder) |
| Non hodgkin lymphoma (disorder) |
| [RFC] Hodgkin's disease (finding) |
| Angioimmunoblastic T-cell lymphoma with dysproteinaemia (disorder) |
| Follicular lymphoma, cutaneous follicle centre (disorder) |
| B-cell non-Hodgkin's lymphoma (disorder) |

| **CNS and miscellaneous intracranial and intraspinal neoplasms** |
| --- |
| Fibrous meningioma (morphologic abnormality) |
| Subependymal giant cell astrocytoma (morphologic abnormality) |
| Myxopapillary ependymoma (morphologic abnormality) |
| Oligodendroglioma, anaplastic (morphologic abnormality) |
| Subependymal glioma (morphologic abnormality) |
| Basophil adenoma (morphologic abnormality) |
| Polar spongioblastoma (morphologic abnormality) |
| Meningeal sarcomatosis (morphologic abnormality) |
| Cerebellar sarcoma (morphologic abnormality) |
| Choroid plexus papilloma, no International Classification of Diseases for Oncology subtype (morphologic abnormality) |
| Meningioma, benign, no International Classification of Diseases for Oncology subtype (morphologic abnormality) |
| Acidophil adenoma (morphologic abnormality) |
| Ependymoma, anaplastic (morphologic abnormality) |
| Mixed acidophil-basophil carcinoma (morphologic abnormality) |
| Medullomyoblastoma (morphologic abnormality) |
| Gliomatosis cerebri (morphologic abnormality) |
| Desmoplastic medulloblastoma (morphologic abnormality) |
| Gliosarcoma (morphologic abnormality) |
| Hemangioblastic meningioma (morphologic abnormality) |
| Hemangiopericytoma (morphologic abnormality) |
| Chromophobe adenoma (morphologic abnormality) |
| Psammomatous meningioma (morphologic abnormality) |
| Astrocytoma, no International Classification of Diseases for Oncology subtype (morphologic abnormality) |
| Primitive neuroectodermal tumor (morphologic abnormality) |
| Craniopharyngioma (morphologic abnormality) |
| Giant cell glioblastoma (morphologic abnormality) |
| Basophil carcinoma (morphologic abnormality) |
| Mixed acidophil-basophil adenoma (morphologic abnormality) |
| Astroblastoma (morphologic abnormality) |
| Acidophil carcinoma (morphologic abnormality) |
| Protoplasmic astrocytoma (morphologic abnormality) |
| Astrocytoma, anaplastic (morphologic abnormality) |
| Clear cell meningioma (morphologic abnormality) |
| Glioblastoma, no International Classification of Diseases for Oncology subtype (morphologic abnormality) |
| Transitional meningioma (morphologic abnormality) |
| Meningothelial meningioma (morphologic abnormality) |
| Fibrillary astrocytoma (morphologic abnormality) |
| Peripheral neuroectodermal tumor (morphologic abnormality) |
| Angiomatous meningioma (morphologic abnormality) |
| Gemistocytic astrocytoma (morphologic abnormality) |
| Glioma, malignant (morphologic abnormality) |
| Meningioma, malignant (morphologic abnormality) |
| Pleomorphic xanthoastrocytoma (morphologic abnormality) |
| Oligodendroblastoma (morphologic abnormality) |
| Malignant rhabdoid tumor (morphologic abnormality) |
| Medulloblastoma, no International Classification of Diseases for Oncology subtype (morphologic abnormality) |
| Choroid plexus carcinoma (morphologic abnormality) |
| Benign neoplasm of craniopharyngeal duct (disorder) |
| Benign neoplasm of pineal gland (disorder) |
| Benign neoplasm of pituitary gland (disorder) |
| Carcinoma in situ of pituitary gland (disorder) |
| Neoplasm of uncertain behavior of craniopharyngeal duct (disorder) |
| Neoplasm of uncertain behavior of pineal gland (disorder) |
| Neoplasm of uncertain behavior of pituitary gland (disorder) |
| Glioma (morphologic abnormality) |
| Neoplasm of pituitary gland (disorder) |
| Meningeal neoplasm (morphologic abnormality) |
| Pituitary carcinoma (morphologic abnormality) |
| Papillary ependymoma (morphologic abnormality) |
| Pilocytic astrocytoma (morphologic abnormality) |
| Central neurocytoma (morphologic abnormality) |
| Malignant tumor of choroid plexus (disorder) |
| Malignant neoplasm of medulla oblongata (disorder) |
| Malignant neoplasm of midbrain (disorder) |
| Cerebral meningioma (disorder) |
| Spinal meningioma (disorder) |
| Craniopharyngioma (disorder) |
| Benign tumor of sella turcica (disorder) |
| Primitive polar spongioblastoma (morphologic abnormality) |
| Monstrocellular sarcoma (morphologic abnormality) |
| Mixed oligoastrocytoma (morphologic abnormality) |
| Pituitary adenoma (disorder) |
| Benign neoplasm of pituitary gland and craniopharyngeal duct (disorder) |
| Malignant tumor of pineal gland (disorder) |
| Ependymoma - category (morphologic abnormality) |
| Oligodendroglioma - category (morphologic abnormality) |
| [M]Meningiomas (morphologic abnormality) |

| **Bone, Soft Tissue and Extraosseous Sarcomas** | |
| --- | --- |
| Fibromyxosarcoma (morphologic abnormality) | |
| Desmoplastic fibroma (morphologic abnormality) | |
| Fibromyxoma (morphologic abnormality) | |
| Sarcomatosis (morphologic abnormality) | |
| Elastofibroma (morphologic abnormality) | |
| Spindle cell sarcoma (morphologic abnormality) | |
| Giant cell tumor of bone, malignant (morphologic abnormality) | |
| Mixed liposarcoma (morphologic abnormality) | |
| Granular cell tumor (morphologic abnormality) | |
| Small cell osteosarcoma (morphologic abnormality) | |
| Clear cell sarcoma (except of Kidney M-89643) (morphologic abnormality) | |
| Fibroblastic osteosarcoma (morphologic abnormality) | |
| Granular cell tumor, malignant (morphologic abnormality) | |
| Mast cell sarcoma (morphologic abnormality) | |
| Embryonal rhabdomyosarcoma (morphologic abnormality) | |
| Chondrosarcoma, no International Classification of Diseases for Oncology subtype (morphologic abnormality) | |
| Myxoid leiomyosarcoma (morphologic abnormality) | |
| Synovial sarcoma, biphasic (morphologic abnormality) | |
| Fascial fibrosarcoma (morphologic abnormality) | |
| Ameloblastic odontosarcoma (morphologic abnormality) | |
| Myosarcoma (morphologic abnormality) | |
| Osteosarcoma, no International Classification of Diseases for Oncology subtype (morphologic abnormality) | |
| Clear cell sarcoma of kidney (morphologic abnormality) | |
| Benign fibrous histiocytoma (morphologic abnormality) | |
| Juxtacortical chondrosarcoma (morphologic abnormality) | |
| Atypical fibrous histiocytoma (morphologic abnormality) | |
| Ameloblastic fibrosarcoma (morphologic abnormality) | |
| Myxoid liposarcoma (morphologic abnormality) | |
| Liposarcoma, well differentiated (morphologic abnormality) | |
| Rhabdomyosarcoma (morphologic abnormality) | |
| Adenosarcoma (morphologic abnormality) | |
| Osteosarcoma in Paget's disease of bone (morphologic abnormality) | |
| Fibrous histiocytoma, malignant (morphologic abnormality) | |
| Synovial sarcoma, spindle cell (morphologic abnormality) | |
| Epithelioid leiomyosarcoma (morphologic abnormality) | |
| Round cell liposarcoma (morphologic abnormality) | |
| Abdominal fibromatosis (morphologic abnormality) | |
| Aggressive fibromatosis (morphologic abnormality) | |
| Soft tissue tumor, benign (morphologic abnormality) | |
| Liposarcoma, no International Classification of Diseases for Oncology subtype (morphologic abnormality) | |
| Kaposi's sarcoma, morphology (morphologic abnormality) | |
| Infantile fibrosarcoma (morphologic abnormality) | |
| Fascial fibroma (morphologic abnormality) | |
| Periosteal fibroma (morphologic abnormality) | |
| Fibrosarcoma (morphologic abnormality) | |
| Fibrous mesothelioma, malignant (morphologic abnormality) | |
| Osteoblastoma (morphologic abnormality) | |
| Synovial sarcoma, epithelioid cell (morphologic abnormality) | |
| Mesenchymal chondrosarcoma (morphologic abnormality) | |
| Epithelioid sarcoma (morphologic abnormality) | |
| Embryonal sarcoma (morphologic abnormality) | |
| Mixed type rhabdomyosarcoma (morphologic abnormality) | |
| Synovial sarcoma (morphologic abnormality) | |
| Carcinosarcoma (morphologic abnormality) | |
| Alveolar rhabdomyosarcoma (morphologic abnormality) | |
| Periosteal fibrosarcoma (morphologic abnormality) | |
| Dedifferentiated liposarcoma (morphologic abnormality) | |
| Endometrial stromal sarcoma, high grade (morphologic abnormality) | |
| Osteoid osteoma (morphologic abnormality) | |
| Small cell sarcoma (morphologic abnormality) | |
| Myxoid chondrosarcoma (morphologic abnormality) | |
| Chondroblastic osteosarcoma (morphologic abnormality) | |
| Dermatofibrosarcoma (morphologic abnormality) | |
| Ewing's sarcoma (morphologic abnormality) | |
| Pleomorphic rhabdomyosarcoma (morphologic abnormality) | |
| Telangiectatic osteosarcoma (morphologic abnormality) | |
| Malignant rhabdoid tumor (morphologic abnormality) | |
| Osteoma, no International Classification of Diseases for Oncology subtype (morphologic abnormality) | |
| Giant cell sarcoma (except of Bone, M-92503) (morphologic abnormality) | |
| Alveolar soft part sarcoma (morphologic abnormality) | |
| Kaposi's sarcoma (disorder) | |
| Kaposi's sarcoma of skin (disorder) | |
| Kaposi's sarcoma of palate (disorder) | |
| Kaposi's sarcoma of multiple organs (disorder) | |
| Angiosarcoma of liver (disorder) | |
| Fibroma, no International Classification of Diseases for Oncology subtype (morphologic abnormality) | |
| Pleomorphic liposarcoma (morphologic abnormality) | |
| Carcinosarcoma, embryonal (morphologic abnormality) | |
| Soft tissue tumor AND/OR sarcoma (morphologic abnormality) | |
| Fibromatous neoplasm (morphologic abnormality) | |
| Osseous AND/OR chondromatous neoplasm (morphologic abnormality) | |
| Granular cell tumor AND/OR alveolar soft part sarcoma (morphologic abnormality) | |
| Parosteal osteosarcoma (morphologic abnormality) | |
| Angiosarcoma of spleen (disorder) | |
| Fibrosarcoma of spleen (disorder) | |
| Kaposi's sarcoma of soft tissue (disorder) | |
| Sarcoma of liver (disorder) | |
| Sarcoma of bone and connective tissue (disorder) |  |
| Histiocytoma (morphologic abnormality) |  |
| Osteosarcoma of bone (disorder) |  |
| Sarcoma - category (morphologic abnormality) |  |
| Fibrohistiocytic neoplasm - category (morphologic abnormality) |  |
| Leiomyosarcoma - category (morphologic abnormality) |  |

| **Renal Tumours** |
| --- |
| Nephroblastoma (morphologic abnormality) |
| Adenosarcoma (morphologic abnormality) |
| Renal cell carcinoma (morphologic abnormality) |
| Adenoma AND/OR adenocarcinoma (morphologic abnormality) |
| Complex mixed AND/OR stromal neoplasm (morphologic abnormality) |
| Neoplasm of kidney (disorder) |
| Juxtaglomerular tumor (morphologic abnormality) |
| Malignant tumor of renal calyx (disorder) |
| Malignant tumor of pelviureteric junction (disorder) |
| Nephroblastoma (disorder) |
| Malignant tumor of renal pelvis (disorder) |
| Malignant tumor of kidney (disorder) |

| **Neuroblastoma and other peripheral nervous system tumours** |
| --- |
| Paraganglioma (morphologic abnormality) |
| Glomangioma (morphologic abnormality) |
| Paraganglioma, malignant (morphologic abnormality) |
| Glomus tumor (morphologic abnormality) |
| Glomangiosarcoma (morphologic abnormality) |
| Malignant pheochromocytoma (morphologic abnormality) |
| Carotid body tumor (morphologic abnormality) |
| Sympathetic paraganglioma (morphologic abnormality) |
| Glomus jugulare tumor (morphologic abnormality) |
| Extra-adrenal paraganglioma, malignant (morphologic abnormality) |
| Glomangiomyoma (morphologic abnormality) |
| Medulloepithelioma (morphologic abnormality) |
| Extra-adrenal paraganglioma (morphologic abnormality) |
| Parasympathetic paraganglioma (morphologic abnormality) |
| Aortic body tumor (morphologic abnormality) |
| Spongioneuroblastoma (morphologic abnormality) |
| Ganglioneuroblastoma (morphologic abnormality) |
| Gangliocytic paraganglioma (morphologic abnormality) |
| Esthesioneuroblastoma (morphologic abnormality) |
| Pheochromocytoma (morphologic abnormality) |
| Neuroblastoma (morphologic abnormality) |
| Teratoid medulloepithelioma (morphologic abnormality) |
| Ganglioglioma, no International Classification of Diseases for Oncology subtype (morphologic abnormality) |
| Paraganglioma AND/OR glomus tumor (morphologic abnormality) |
| Sympathicoblastoma (morphologic abnormality) |

| **Hepatic Tumours** |
| --- |
| Hepatocellular carcinoma (morphologic abnormality) |
| Hepatoblastoma (morphologic abnormality) |
| Carcinoma in situ of ampulla of Vater (disorder) |
| Carcinoma in situ of biliary tract (disorder) |
| Carcinoma in situ of common bile duct (disorder) |
| Carcinoma in situ of cystic duct (disorder) |
| Carcinoma in situ of gallbladder (disorder) |
| Carcinoma in situ of intrahepatic bile ducts (disorder) |
| Carcinoma in situ of liver (disorder) |
| Liver cell carcinoma (disorder) |
| Hepatoblastoma (disorder) |
| Primary carcinoma of liver (disorder) |
| Carcinoma in situ of hepatic duct (disorder) |
| Carcinoma in situ of sphincter of Oddi (disorder) |
| Carcinoma in situ of liver and/or biliary system (disorder) |
| [RFC] Liver cancer (finding) |

| **Gonadal germ cell tumours (21)** |
| --- |
| Spermatocytic seminoma (morphologic abnormality) |
| Struma ovarii, malignant (morphologic abnormality) |
| Teratoma, malignant, no International Classification of Diseases for Oncology subtype (morphologic abnormality) |
| Malignant teratoma, intermediate (morphologic abnormality) |
| Struma ovarii (morphologic abnormality) |
| Embryonal carcinoma (morphologic abnormality) |
| Germinoma (morphologic abnormality) |
| Polyembryoma (morphologic abnormality) |
| Strumal carcinoid (morphologic abnormality) |
| Mixed germ cell tumor (morphologic abnormality) |
| Teratoma, benign (morphologic abnormality) |
| Teratoma, no International Classification of Diseases for Oncology subtype (morphologic abnormality) |
| Malignant dysgerminoma (morphologic abnormality) |
| Teratocarcinoma (morphologic abnormality) |
| Seminoma, anaplastic (morphologic abnormality) |
| Endodermal sinus tumor (morphologic abnormality) |
| Gonadoblastoma (morphologic abnormality) |
| Malignant teratoma, undifferentiated (morphologic abnormality) |
| Germ cell neoplasm (morphologic abnormality) |
| Dermoid cyst with malignant transformation (morphologic abnormality) |
| Seminoma - category (morphologic abnormality) |

| **Supplementary Table 2. Clinical features associated with CTYA cancer per cancer subtype** | | | | | | |
| --- | --- | --- | --- | --- | --- | --- |
| **Clinical Features** | **Leukaemias** | **Lymphomas** | **CNS Tumours** | **Sarcomas** | **Abdominal Tumours** | **Gonadal Germ Cell Tumours** |
| **Systemic / Endocrine** | Fever  Weight loss  Anorexia  Night sweats  Lethargy/tiredness  Feels unwell | Fever  Weight loss  Anorexia  Night sweats  Lethargy/tiredness  Pruritus  Feels unwell | Fever  Weight loss  Anorexia  Lethargy/tiredness  Irritability  Developmental delay  Precocious puberty  Feels unwell | Fever  Weight loss  Anorexia  Lethargy/tiredness  Feels unwell | Fever  Weight loss  Anorexia  Lethargy/tiredness  Feels unwell | Fever  Weight loss  Anorexia  Lethargy/tiredness  Menstrual abnormalities  Precocious puberty  Feels unwell |
| **Masses** | Lymphadenopathy  Swelling (head or body)  Lump (head or body)  Organomegaly  Hepatomegaly  Splenomegaly  Hepatosplenomegaly  Abdominal mass | Lymphadenopathy  Swelling (head or body)  Lump (head or body)  Organomegaly  Hepatomegaly  Splenomegaly  Hepatosplenomegaly  Abdominal mass | - | Swelling (head or body)  Lump (head or body)  Abdominal mass | Abdominal mass | Abdominal mass  Testicular mass/swelling |
| **Cardiorespiratory** | Cough  Chest pain  Breathlessness  URTI/sore throat | Cough  Chest pain  Breathlessness  Stridor  URTI/sore throat | - | Cough  Chest pain  Breathlessness | - | Chest pain |
| **Gastrointestinal** | Abdominal pain  Vomiting | Abdominal pain  Vomiting | Vomiting | Abdominal pain  Vomiting  Constipation  Haematuria | Abdominal pain  Vomiting  Constipation  Haematuria | Abdominal pain  Abdominal distension  Vomiting  Constipation  Haematuria |
| **Neurological** | Headache  Dizziness  Loss of consciousness | Headache  Dizziness  Loss of consciousness | Headache  Dizziness  Loss of consciousness  Unsteady gait  Squint  Visual acuity problems  Abnormal eye movements  Diplopia  Papilloedema  Optic atrophy  Exopthalmos  Hemiparesis  Hemiplegia  Macrocephaly/large head  Seizures  Torticollis  Voice changes  Cranial nerve palsies | Headache | Headache  Cranial nerve palsies  Unsteady gait |  |
| **Haematological** | Bruising/bleeding  Anaemia/pallor | Anaemia/pallor | - | - | - |  |
| **Musculoskeletal** | Limb pain  Joint pain  Limp | Limb pain  Joint pain | Limp | Limb pain  Joint pain  Limp | Limb pain  Joint pain  Limp |  |
| **Dermatological** | Rash  Skin nodules/infiltrations | Rash  Skin nodules/infiltrations | - | Rash  Skin nodules/infiltrations | - |  |

**Supplementary Table 3 STROBE Checklist**

|  | **Item No** | **Recommendation** |
| --- | --- | --- |
| **Title and abstract**  Pag 1-2 | 1 | (*a*) Indicate the study’s design with a commonly used term in the title or the abstract |
|  |  | (*b*) Provide in the abstract an informative and balanced summary of what was done and what was found |
| **Introduction** | | |
| Background/rationale  Pag 3 | 2 | Explain the scientific background and rationale for the investigation being reported |
| Objectives   Pag 3 | 3 | State specific objectives, including any prespecified hypotheses |
| **Methods** | | |
| Study design   Pag 4 | 4 | Present key elements of study design early in the paper |
| Setting  Pag 4 | 5 | Describe the setting, locations, and relevant dates, including periods of recruitment, exposure, follow-up, and data collection |
| Participants  Pag 4 | 6 | (*a*) Give the eligibility criteria, and the sources and methods of selection of participants. Describe methods of follow-up |
|  |  | (*b*) For matched studies, give matching criteria and number of exposed and unexposed |
| Variables  Pag 4-5 | 7 | Clearly define all outcomes, exposures, predictors, potential confounders, and effect modifiers. Give diagnostic criteria, if applicable |
| Data sources/ measurement  Pag 4 | 8* | For each variable of interest, give sources of data and details of methods of assessment (measurement). Describe comparability of assessment methods if there is more than one group |
| Bias  Pag 5 | 9 | Describe any efforts to address potential sources of bias |
| Study size  N/A includes all population | 10 | Explain how the study size was arrived at |
| Quantitative variables  Page 5 | 11 | Explain how quantitative variables were handled in the analyses. If applicable, describe which groupings were chosen and why |
| Statistical methods  Page 5-6 | 12 | (*a*) Describe all statistical methods, including those used to control for confounding |
|  |  | (*b*) Describe any methods used to examine subgroups and interactions |
|  |  | (*c*) Explain how missing data were addressed |
|  |  | (*d*) If applicable, explain how loss to follow-up was addressed |
|  |  | (*e*) Describe any sensitivity analyses |
| **Results** | | |
| Participants  Pag 6-7 | 13* | (a) Report numbers of individuals at each stage of study—eg numbers potentially eligible, examined for eligibility, confirmed eligible, included in the study, completing follow-up, and analysed |
|  |  | (b) Give reasons for non-participation at each stage |
|  |  | (c) Consider use of a flow diagram |
| Descriptive data  Pag 6-7 | 14* | (a) Give characteristics of study participants (eg demographic, clinical, social) and information on exposures and potential confounders |
|  |  | (b) Indicate number of participants with missing data for each variable of interest |
|  |  | (c) Summarise follow-up time (eg, average and total amount) |
| Outcome data  Table 1 | 15* | Report numbers of outcome events or summary measures over time |
| Main results  Pag 7-9 and Tables 2,3, Fig 1-4 | 16 | (*a*) Give unadjusted estimates and, if applicable, confounder-adjusted estimates and their precision (eg, 95% confidence interval). Make clear which confounders were adjusted for and why they were included |
|  |  | (*b*) Report category boundaries when continuous variables were categorized |
|  |  | (*c*) If relevant, consider translating estimates of relative risk into absolute risk for a meaningful time period |
| Other analyses  supplementary appendix | 17 | Report other analyses done—eg analyses of subgroups and interactions, and sensitivity analyses |
| **Discussion** | | |
| Key results  Pag 10 | 18 | Summarise key results with reference to study objectives |
| Limitations  Pag 12 | 19 | Discuss limitations of the study, taking into account sources of potential bias or imprecision. Discuss both direction and magnitude of any potential bias |
| Interpretation  Pag 10-12 | 20 | Give a cautious overall interpretation of results considering objectives, limitations, multiplicity of analyses, results from similar studies, and other relevant evidence |
| Generalisability  Pag 11-12 | 21 | Discuss the generalisability (external validity) of the study results |
| **Other information** | | |
| Funding  Pag 15 | 22 | Give the source of funding and the role of the funders for the present study and, if applicable, for the original study on which the present article is based |

*Give information separately for exposed and unexposed groups.

**Note:** An Explanation and Elaboration article discusses each checklist item and gives methodological background and published examples of transparent reporting. The STROBE checklist is best used in conjunction with this article (freely available on the Web sites of PLoS Medicine at http://www.plosmedicine.org/, Annals of Internal Medicine at http://www.annals.org/, and Epidemiology at http://www.epidem.com/). Information on the STROBE Initiative is available at http://www.strobe-statement.org.

*Supplementary Table 4. Minimum sample size required to detect an odds ratio of 2 for a single feature separately for childhood (0-14 years) and TYA (15-24 years). 95% CI= 95% Confidence Interval. The probability of exposure in the control population has been derived by two primary care based studies carried out by Dommett and colleagues^1,2^.*

| **Cancer Type** | **Clinical Feature** | **Age Group** | **Minimum sample size**  **Required to detect an odds ratio of 2 for my proposed study** α**=0.05 and 1-**β**=0.8** |
| --- | --- | --- | --- |
| Any | Fatigue | 0-14 years | 5295 |
|  |  | 15-24 years | 2660 |
| Leukaemia | Lymphadenopathy | 0-14 years | 3538 |
|  |  | 15-24 years | 5295 |
| Lymphoma | Lymphadenopathy | 0-14 years | 3538 |
|  |  | 15-24 years | 5295 |
| CNS  Tumours | Headache | 0-14 years | 2133 |
|  |  | 15-24 years | 1781 |
| Sarcoma | Musculoskeletal symptoms | 0-14 years | 1079 |
|  |  | 15-24 years | 1079 |

**References:**

1.Dommett RM, Redaniel MT, Stevens MC, Hamilton W, Martin RM. Features of childhood cancer in primary care: a population-based nested case-control study. *Br J Cancer* 2012; **106**(5): 982-7.

2.Dommett RM, Redaniel MT, Stevens MC, Hamilton W, Martin RM. Features of cancer in teenagers and young adults in primary care: a population-based nested case-control study. *Br J Cancer* 2013; **108**(11): 2329-33.

**Supplementary Table 5. SNOMED CT Definitions**

Gastrointestinal Symptoms/Signs

| **Abdominal Mass (SNOMED Fully Specified Name)** |
| --- |
| On examination - abdominal mass palpated (finding) |
| On examination - abdominal mass less than 1 quadrant (finding) |
| On examination - abdominal mass fills 1 quadrant (finding) |
| On examination - abdominal mass fills half abdomen (finding) |
| On examination - abdominal mass fills abdomen (finding) |
| Right iliac fossa mass (finding) |
| On examination - abdominal mass consistency (finding) |
| On examination - abdominal mass - soft (finding) |
| On examination - abdominal mass - hard (finding) |
| On examination - abdominal mass-very hard (finding) |
| On examination - pulsatile mass of abdomen (finding) |
| On examination - abdominal mass shape (finding) |
| On examination - abdominal mass - regular shape (finding) |
| On examination - abdominal mass - irregular shape (finding) |
| On examination - abdominal mass movement with respiration (finding) |
| On examination - abdominal mass still with respiration (finding) |
| On examination - abdominal mass -border defined (finding) |
| On examination - abdominal mass - upper border defined (finding) |
| On examination - abdominal mass - lower border defined (finding) |
| On examination - tympany over abdominal mass (finding) |
| On examination - dullness over abdominal mass (finding) |
| On examination - pelvic mass palpated (finding) |
| On examination - pelvic mass palpable-left iliac fossa (finding) |
| On examination - pelvic mass palpable-right iliac fossa (finding) |
| On examination - central pelvic mass (finding) |
| Abdominal mass (finding) |
| Intra-abdominal and pelvic swelling, mass and lump (finding) |
| Left iliac fossa mass (finding) |
| Epigastric mass (finding) |
| On examination - left lower abdominal mass (finding) |
| Navigational concept (navigational concept) |
| Central abdominal mass (finding) |
| Right upper quadrant mass (finding) |
| Left upper quadrant mass (finding) |
| Right flank mass (finding) |
| Left flank mass (finding) |

| **Abdominal Pain (SNOMED Fully Specified Name)** |
| --- |
| Acute abdomen (disorder) |
| Abdominal colic (finding) |
| Abdominal pain (finding) |
| Infantile colic (finding) |
| Abdominal tenderness (finding) |
| Abdominal wind pain (finding) |
| Stomach cramps (finding) |
| Lower abdominal pain (finding) |
| Abdominal migraine (disorder) |
| Epigastric pain (finding) |
| Upper abdominal pain (finding) |
| Umbilical pain (finding) |
| Inguinal pain (finding) |
| Generalized abdominal pain (finding) |
| Non-colicky abdominal pain (finding) |
| Abdominal wall pain (finding) |
| Central abdominal pain (finding) |
| Left flank pain (finding) |
| Right flank pain (finding) |
| Right iliac fossa pain (finding) |
| Left iliac fossa pain (finding) |
| Suprapubic pain (finding) |
| Complaining of pelvic pain (finding) |
| Evening colic (finding) |
| Loin pain (finding) |
| Recurrent acute abdominal pain (finding) |
| Pelvic and perineal pain (finding) |
| Left upper quadrant pain (finding) |
| Left lower quadrant pain (finding) |
| Right upper quadrant pain (finding) |
| Right lower quadrant pain (finding) |
| Nonspecific abdominal pain (finding) |
| On examination - epigastric pain on palpation (finding) |
| Left loin pain (finding) |
| Groin pain (finding) |
| Colicky abdominal pain present (finding) |
| Manchester triage - Abdominal pain in child (finding) |

| **Organomegaly (SNOMED Fully Specified Name)** |
| --- |
| Large liver (disorder) |
| On examination - liver edge palpable (finding) |
| On examination - liver moderately enlarged (disorder) |
| On examination - liver grossly enlarged (disorder) |
| On examination - liver character (finding) |
| On examination - liver soft (finding) |
| On examination - liver firm (finding) |
| On examination - liver very hard (finding) |
| On examination - liver grossly irregular (finding) |
| On examination - liver finely irregular (finding) |
| On examination - liver smooth (finding) |
| On examination - liver tender (finding) |
| On examination - liver pulsatile (finding) |
| On examination - liver palpated (finding) |
| On examination - hepatomegaly (disorder) |
| Splenomegaly (disorder) |
| On examination - splenomegaly (finding) |
| On examination - spleen just palpable (finding) |
| On examination - moderate splenomegaly (disorder) |
| On examination - gross splenomegaly (disorder) |
| On examination - painful splenomegaly (disorder) |
| Hepatosplenomegaly (disorder) |

| **Vomiting/nausea (SNOMED Fully Specified Name)** |
| --- |
| Projectile vomiting (disorder) |
| Hematemesis (disorder) |
| Nausea and vomiting (disorder) |
| Cyclical vomiting syndrome (disorder) |
| Rumination (finding) |
| Coffee ground vomiting (disorder) |
| Habit vomiting (disorder) |
| Morning sickness (finding) |
| Bilious vomiting (disorder) |
| Retching (finding) |
| Nausea present (situation) |
| Vomit examination (procedure) |
| Persistent vomiting (disorder) |
| Posseting (finding) |
| Vomiting symptom (finding) |
| Vomiting blood - fresh (disorder) |
| Vomit: frank blood present (finding) |
| Bilious vomit (finding) |
| Complaining of vomiting (finding) |
| Bilious vomit on examination (disorder) |
| Blood in vomit on examination (finding) |
| Blood in vomit - symptom (finding) |
| Finding of vomiting (finding) |
| Vomitus with coffee ground appearance (finding) |
| Drug-induced nausea and vomiting (disorder) |
| Vomiting (disorder) |
| Nausea (finding) |
| Frequency of vomiting (observable entity) |
| Nausea/vomiting (finding) |
| Reason for referral: Vomiting/Nausea (finding) |
| 24 hour vomitus output (finding) |

| **Abdominal Distension (SNOMED Fully Specified Name)** |
| --- |
| Abdominal distension symptom (finding) |
| Bloating symptom (finding) |
| Abdominal distension, gaseous (finding) |

Genitourinary Symptoms/Signs

| **Testicular abnormalities** |
| --- |
| Testicular mass (finding) |
| Testicular swelling (finding) |

| **Female Reproductive System** |
| --- |
| Menorrhagia (finding) |
| Heavy episode of vaginal bleeding (finding) |
| Complaining of per vaginam bleeding (finding) |
| Bleeding from vagina (finding) |
| Abnormal vaginal bleeding (finding) |
| Heavy episode of vaginal bleeding (finding) |

Cardiorespiratory Symptoms/Signs

| **(SNOMED Fully Specified Name)** |
| --- |
| Pleuritic pain (finding) |
| Retrosternal pain (finding) |
| Dry cough (finding) |
| Barking cough (finding) |
| Tight chest (finding) |
| Productive cough (finding) |
| Chest pain (finding) |
| Cough (finding) |
| Dyspnea on exertion (finding) |
| Orthopnea (finding) |
| Hemoptysis (finding) |
| Chronic cough (finding) |
| Breathing painful (finding) |
| Chest pain on exertion (finding) |
| Chest wall pain (finding) |
| Atypical chest pain (finding) |
| Productive cough -clear sputum (finding) |
| Productive cough -green sputum (finding) |
| Productive cough-yellow sputum (finding) |
| Night cough present (situation) |
| Night cough absent (situation) |
| Chesty cough (finding) |
| Morning cough (finding) |
| Evening cough (finding) |
| Breathless - moderate exertion (finding) |
| Breathless - mild exertion (finding) |
| Dyspnea at rest (finding) |
| Nocturnal cough / wheeze (finding) |
| Central chest pain (finding) |
| Anterior chest wall pain (finding) |
| Parasternal pain (finding) |
| Costal margin chest pain (finding) |
| On examination - dyspnea (finding) |
| On examination - expiratory wheeze (finding) |
| Difficulty breathing (finding) |
| Dyspnea (finding) |
| Complaining of cough (finding) |
| Non-cardiac chest pain (finding) |
| Chest discomfort (finding) |
| Musculoskeletal chest pain (finding) |
| Persistent cough (finding) |
| Unexplained cough (finding) |
| Short of breath dressing/undressing (finding) |
| Breathless - strenuous exertion (finding) |
| Unable to complete a sentence in one breath (finding) |
| Difficulty eating due to breathlessness (finding) |
| On examination - inspiratory wheeze (finding) |
| Viral wheeze (disorder) |
| Mild wheeze (finding) |
| Moderate wheeze (finding) |
| Severe wheeze (finding) |
| Very severe wheeze (finding) |
| Episodic dry cough (finding) |
| H/O: chest pain (situation) |
| Dyspnoea (finding) |
| O/E - dyspnoea at rest (finding) |
| O/E - dyspnoea on exertion (finding) |
| Constant wheeze (finding) |
| Breathlessness causing difficulty eating (finding) |
| Breathlessness causing anxiety (finding) |
| Manchester triage - Shortness of breath in child (finding) |

Neurological Symptoms/Signs

| **Papilloedema (SNOMED Fully Specified Name)** |
| --- |
| On examination - optic disc margin blurred (finding) |
| On examination - optic disc margin absent (finding) |
| On examination - optic disc elevated (finding) |
| Edema of optic disc (disorder) |
| Papilledema - optic disc edema due to raised intracranial pressure (disorder) |
| Optic disc edema due to hypotony (disorder) |
| Optic disc edema associated with retinal disorder (disorder) |

| **Headache (SNOMED Fully Specified Name)** |
| --- |
| Occipital headache (finding) |
| Migraine with aura (disorder) |
| Sinus headache (finding) |
| Headache (finding) |
| Migraine (disorder) |
| Temporal headache (finding) |
| Recurrent painful ophthalmoplegic neuropathy (disorder) |
| Migraine without aura (disorder) |
| Hemiplegic migraine (disorder) |
| Abdominal migraine (disorder) |
| Basilar migraine (disorder) |
| Chronic paroxysmal hemicrania (disorder) |
| Ophthalmic migraine (disorder) |
| Unilateral headache (situation) |
| History of migraine (situation) |
| Headache site (finding) |
| Generalized headache (finding) |
| Bilateral headache (finding) |
| Parietal headache (finding) |
| Headache character (observable entity) |
| Aching headache (finding) |
| Throbbing headache (finding) |
| Shooting headache (finding) |
| Morning headache (finding) |
| Heavy head (finding) |
| Sick headache (disorder) |
| Migraine variants (disorder) |
| Cluster headache syndrome (disorder) |
| Complicated migraine (disorder) |
| Migraine with typical aura (disorder) |
| Status migrainosus (disorder) |
| Chronic tension-type headache (disorder) |
| Frontal headache (finding) |
| Complaining of a headache (finding) |
| Finding of headache character (finding) |
| Tension-type headache (disorder) |
| Muscular headache (disorder) |
| Chronic headache disorder (disorder) |
| Paroxysmal hemicrania (disorder) |
| Hemicrania continua (disorder) |
| Trigeminal autonomic cephalalgia (disorder) |
| Infrequent episodic tension-type headache (disorder) |
| Frequent episodic tension-type headache (disorder) |
| Short-lasting unilateral neuralgiform headache attacks with conjunctival injection and tearing syndrome (disorder) |
| [D]Pain in head NOS (situation) |
| Migraine induced by oestrogen contraceptive (disorder) |
| Chronic headache disorder (disorder) |
| Chronic Paroxysmal Hemicrania (disorder) |
| Unexplained recurrent headaches (finding) |

| **Focal Motor Weakness (SNOMED Fully Specified Name)** |
| --- |
| Hemiparesis (disorder) |
| Muscle weakness (finding) |
| Weakness present (finding) |
| On examination - paresis (weakness) (finding) |
| Monoparesis - arm (disorder) |
| Monoparesis of lower limb (disorder) |
| Right hemiparesis (disorder) |
| Left hemiparesis (disorder) |
| Hemiparesis (finding) |
| Right hemiparesis (disorder) |
| Left hemiparesis (finding) |
| Weakness of upper arm (disorder) |
| Weakness of lower arm (disorder) |

| **Squint (SNOMED Fully Specified Name)** |
| --- |
| Intermittent vertical heterotropia (disorder) |
| Monocular esotropia (disorder) |
| Monocular exotropia (disorder) |
| Esotropia (disorder) |
| Vertical heterophoria (disorder) |
| Strabismus (disorder) |
| Alternating esotropia with V pattern (disorder) |
| Intermittent esotropia (disorder) |
| Microstrabismus (disorder) |
| Hypotropia (disorder) |
| Alternating exotropia with A pattern (disorder) |
| Alternating exotropia (disorder) |
| Alternating esotropia (disorder) |
| Hypertropia (disorder) |
| Monocular exotropia with A pattern (disorder) |
| Exophoria (disorder) |
| Alternating exotropia with V pattern (disorder) |
| Monocular esotropia with A pattern (disorder) |
| Alternating hyperphoria (disorder) |
| Cyclophoria (disorder) |
| Esophoria (disorder) |
| Intermittent exotropia (disorder) |
| Monocular esotropia with V pattern (disorder) |
| Cyclotropia (disorder) |
| Intermittent tropia (disorder) |
| Alternating esotropia with A pattern (disorder) |
| Monocular exotropia with V-pattern strabismus (disorder) |
| Heterotropia (disorder) |
| On examination - strabismus - squint (finding) |
| On examination - convergent squint (disorder) |
| On examination - divergent squint (disorder) |
| Child examination: squint (procedure) |
| Monocular esotropia with X pattern (disorder) |
| Monocular esotropia with Y pattern (disorder) |
| Alternating esotropia with X pattern (disorder) |
| Alternating esotropia with Y pattern (disorder) |
| Monocular exotropia with X pattern (disorder) |
| Monocular exotropia with Y pattern (disorder) |
| Alternating exotropia with X pattern (disorder) |
| Alternating exotropia with Y pattern (disorder) |
| Intermittent monocular esotropia (disorder) |
| Intermittent alternating esotropia (disorder) |
| Intermittent monocular exotropia (disorder) |
| Intermittent alternating exotropia (disorder) |
| Constant vertical heterotropia (disorder) |
| Esotropia with accommodative compensation (disorder) |
| Has a squint (disorder) |
| Exotropia (disorder) |
| Paralytic strabismus (disorder) |
| Strabismus (squint) and other disorders of binocular eye movements (disorder) |
| Possible squint (situation) |
| Possible squint present (finding) |
| Squint present on examination (finding) |
| [RFC] Sudden onset squints/double vision (finding) |
| Childs exam. - squint (navigational concept) |

| **Seizure (SNOMED Fully Specified Name)** |
| --- |
| Focal onset epileptic seizure (finding) |
| Myoclonic seizure (disorder) |
| Tonic-clonic seizure (finding) |
| Absence seizure (finding) |
| Seizure (finding) |
| Atonic epileptic seizure (finding) |
| Epileptic seizures - akinetic (finding) |
| Clonic epileptic seizure (finding) |
| Epileptic seizures - myoclonic (disorder) |
| Tonic epileptic seizure (finding) |
| Seizures in response to acute event (disorder) |
| Nocturnal epilepsy (disorder) |
| Generalized onset epileptic seizure (finding) |
| Focal onset impaired awareness epileptic seizure (finding) |
| Reflex anoxic seizure (finding) |
| Epileptic seizure witnessed by provider of history other than subject (finding) |
| Simple partial epileptic seizure (disorder) |

| **Precocious Puberty (SNOMED Fully Specified Name)** |
| --- |
| Premature adrenarche (finding) |
| Precocious pubarche (disorder) |
| Precocious puberty with adrenal hyperplasia (disorder) |
| Precocious puberty (disorder) |

| **Abnormal visual acuity/vision (SNOMED Fully Specified Name)** |
| --- |
| On examination - visual acuity right eye (finding) |
| On examination - visual acuity right eye =6/9 (finding) |
| On examination - visual acuity right eye=6/12 (finding) |
| On examination - visual acuity right eye=6/18 (finding) |
| On examination - visual acuity right eye=6/24 (finding) |
| On examination - visual acuity right eye=6/36 (finding) |
| On examination - visual acuity right eye=6/60 (finding) |
| On examination - visual acuity left eye =6/9 (finding) |
| On examination - visual acuity left eye=6/12 (finding) |
| On examination - visual acuity left eye=6/18 (finding) |
| On examination - visual acuity left eye=6/24 (finding) |
| On examination - visual acuity left eye=6/36 (finding) |
| On examination - visual acuity left eye=6/60 (finding) |
| On examination - near vision abnormal (finding) |
| Poor visual acuity (finding) |
| Sight deteriorating (finding) |
| On examination - visual acuity right eye=3/60 (finding) |
| On examination - visual acuity left eye=3/60 (finding) |
| On examination - pinhole visual acuity left eye=6/9 (finding) |
| On examination - pinhole visual acuity left eye=6/12 (finding) |
| On examination - pinhole visual acuity left eye=6/18 (finding) |
| On examination - pinhole visual acuity left eye =6/24 (finding) |
| On examination - pinhole visual acuity left eye=6/36 (finding) |
| On examination - pinhole visual acuity left eye=6/60 (finding) |
| On examination - pinhole visual acuity right eye=6/9 (finding) |
| On examination - pinhole visual acuity right eye=6/12 (finding) |
| On examination - pinhole visual acuity right eye=6/18 (finding) |
| On examination - pinhole visual acuity right eye=6/24 (finding) |
| On examination - pinhole visual acuity right eye=6/36 (finding) |
| On examination - pinhole visual acuity right eye=6/60 (finding) |
| On examination - visual acuity right eye =6/15 (finding) |
| On examination - visual acuity left eye =6/15 (finding) |
| On examination - visual acuity left eye = 6/7.5 (finding) |
| On examination - visual acuity left eye = 6/48 (finding) |
| On examination - visual acuity right eye = 6/48 (finding) |
| On examination - visual acuity right eye = 6/7.5 (finding) |
| On examination visual acuity left eye = 6/30 (finding) |
| On examination visual acuity right eye = 6/30 (finding) |
| Loss of vision (disorder) |
| On examination visual acuity left eye = 6/20 (finding) |
| On examination visual acuity right eye = 6/20 (finding) |
| On examination visual acuity right eye = 6/10 (finding) |
| On examination visual acuity left eye = 6/10 (finding) |
| On examination visual acuity left eye = 6/7 (finding) |
| On examination visual acuity right eye = 6/7 (finding) |
| Visual impairment: unable to recognise people across street (disorder) |
| Visual impairment: difficulty with close up work (disorder) |
| Patient reports difficulty with peripheral vision (disorder) |
| Visual impairment: difficulty using stairs (disorder) |
| Visual impairment: avoids driving (disorder) |
| Visual impairment: difficulty reading newspaper print (disorder) |
| Patient concerned about eyesight (disorder) |
| O/E - visual acuity R-eye=6/15 (finding) |
| O/E - visual acuity L-eye=6/15 (finding) |
| O/E - visual acuity L-eye = 6/48 (finding) |
| O/E - visual acuity L-eye = 6/7.5 (finding) |
| O/E - visual acuity R-eye = 6/48 (finding) |
| O/E - visual acuity R-eye = 6/7.5 (finding) |
| O/E - visual acuity R-eye =6/20 (finding) |
| O/E - visual acuity R-eye =6/10 (finding) |
| O/E - visual acuity L-eye =6/20 (finding) |
| O/E - visual acuity L-eye =6/10 (finding) |
| O/E - visual acuity L-eye=6/7 (finding) |
| O/E - visual acuity R-eye=6/7 (finding) |
| Child exam - vision: problem identified (procedure) |
| Child exam - vision: problem suspected (procedure) |

| **Gait abnormalities (SNOMED Fully Specified Name)** |
| --- |
| Spastic gait (finding) |
| Dyskinesia (finding) |
| Ataxia (finding) |
| Abnormal gait (finding) |
| Ataxic gait (finding) |
| Muscular incoordination (finding) |
| Staggering gait (finding) |
| Loss of equilibrium (finding) |
| On examination - gait spastic (finding) |
| On examination - gait ataxic (finding) |
| On examination - waddling gait (finding) |
| On examination - steppage gait (finding) |
| On examination - hysterical gait (finding) |
| On examination - arms ataxic (finding) |
| On examination - legs ataxic (finding) |
| On examination - Romberg test positive (finding) |
| On examination - generally unsteady (finding) |
| On examination - clumsy (finding) |
| Walking difficulty due to pelvic region and thigh (finding) |
| Feels as though will fall (finding) |
| Toe-walking gait (finding) |
| Incoordination symptom (finding) |
| On examination - festination-Parkinson gait (finding) |
| On examination - ataxia (finding) |
| Incoordination (finding) |
| Difficulty walking up stairs (finding) |
| Unable to balance (finding) |
| Difficulty walking (finding) |
| Disorder of gait and/or balance present (situation) |
| Disorder of gait and/or balance not present (situation) |
| [D]Gait abnormality (situation) |
| Worsening balance (finding) |
| Child gait delayed (procedure) |
| Child gait abnormal (procedure) |
| Gait/ambulation disturbance (finding) |
| O/E gait abnormal (finding) |
| CHSP-PS recall: gait abnormal (regime/therapy) |
| CHSP-PS unsched. contact: gait abnormal (regime/therapy) |

Systemic Symptoms/Signs

| **Fever (SNOMED Fully Specified Name)** |
| --- |
| Pyrexia of unknown origin (finding) |
| Feverish cold (finding) |
| On examination - skin temperature abnormal (finding) |
| On examination - fever - general (finding) |
| On examination - pyrexia of unknown origin (finding) |
| On examination - temperature elevated (finding) |
| On examination - hyperpyrexia - greater than 40.5 degrees Celsius (finding) |
| On examination - fever - acute rise (finding) |
| On examination - fever - gradual rise (finding) |
| On examination - fever - continuous (finding) |
| On examination - fever - remittent (finding) |
| On examination - fever - intermittent (finding) |
| On examination - staircase fever (finding) |
| On examination - fever - irregular (finding) |
| On examination - fever - fast fall-crisis (finding) |
| On examination - fever-gradual fall-lysis (finding) |
| Sweating fever (finding) |
| Fever symptoms (finding) |
| Examination of fever (procedure) |
| Temperature symptoms (finding) |
| On examination - fever (finding) |
| Fever with chills (finding) |
| On examination - temperature (finding) |
| Feels hot/feverish (finding) |
| Fever (finding) |
| Hyperpyrexia (finding) |
| [D]Persistent fever (situation) |
| [D]Pyrexia (situation) |
| Fever (finding) |
| Pyrexia (finding) |

| **Weight loss (SNOMED Fully Specified Name)** |
| --- |
| Weight decreasing (finding) |
| Abnormal weight loss (finding) |
| Unintentional weight loss (finding) |
| Complaining of weight loss (finding) |
| Abn. weight/height/head circum. relation to growth/age standards (finding) |
| Unexplained/progressive weight loss (finding) |

| **Lethargy/tiredness (SNOMED Fully Specified Name)** |
| --- |
| Exhaustion (finding) |
| Fatigue (finding) |
| Lethargy (finding) |
| Lack of energy (finding) |
| Tiredness symptom (finding) |
| Tired all the time (finding) |
| Malaise and fatigue (finding) |
| Fatigue - symptom (finding) |
| Complaining of "tired all the time" (finding) |
| Malaise (finding) |

| **Night Sweats (SNOMED Fully Specified Name)** |
| --- |
| Night sweats (finding) |
| Excessive sweating (finding) |
| Sweating symptom (finding) |

Haematological/Masses

| **Lymphadenopathy (SNOMED Fully Specified Name)** |
| --- |
| Inguinal lymph node structure (body structure) |
| Lymphadenopathy (disorder) |
| Pelvic lymph node structure (body structure) |
| Structure of lymph node (body structure) |
| Mediastinal lymph node structure (body structure) |
| Axillary lymph node structure (body structure) |
| Cervical lymph node structure (body structure) |
| Structure of scalene lymph node (body structure) |
| Structure of retroperitoneal lymph node (body structure) |
| Cervical lymphadenopathy (disorder) |
| On examination - sub-mental lymphadenopathy (disorder) |
| On examination - supraclavicular lymphadenopathy (disorder) |
| On examination - axillary lymphadenopathy (disorder) |
| On examination - inguinal lymphadenopathy (disorder) |
| On examination - popliteal lymphadenopathy (disorder) |
| On examination - lymph node character (finding) |
| On examination - lymph nodes firm (finding) |
| On examination - lymph nodes hard (finding) |
| On examination - lymph nodes tethered (finding) |
| On examination - lymph nodes discrete (finding) |
| On examination - lymph nodes fluctuant (finding) |
| On examination - lymph nodes tender (finding) |
| Para-aortic node (body structure) |
| On examination - lymph nodes (finding) |
| Enlarged submandibular lymph gland (disorder) |
| Generalized enlarged lymph nodes (disorder) |
| Localized enlarged lymph nodes (disorder) |
| On examination - post-auricular lymphadenopathy (disorder) |
| Reactive lymphadenopathy (disorder) |
| Other lymph node - OPCS Classification of Interventions and Procedures (body structure) |

| **Bruising (SNOMED Fully Specified Name)** |
| --- |
| Contusion of multiple sites of trunk (disorder) |
| Contusion of cheek (disorder) |
| Contusion of neck (disorder) |
| Contusion of chest (disorder) |
| Contusion of back (disorder) |
| Contusion of toenail (disorder) |
| Contusion of groin (disorder) |
| Contusion of scalp (disorder) |
| Contusion of knee (disorder) |
| Contusion of trunk (disorder) |
| Contusion of abdominal wall (disorder) |
| Contusion of forearm (disorder) |
| Contusion of shoulder region (disorder) |
| Contusion of orbital tissues (disorder) |
| Contusion of hip (disorder) |
| Contusion of lower leg (disorder) |
| Contusion of wrist (disorder) |
| Contusion of ear (disorder) |
| Contusion of mandibular joint area (disorder) |
| Contusion of axillary region (disorder) |
| Contusion of interscapular region (disorder) |
| Contusion of ankle (disorder) |
| Contusion of toe (disorder) |
| Contusion of multiple sites of upper limb (disorder) |
| Contusion of flank (disorder) |
| Contusion of nose (disorder) |
| Contusion of lip (disorder) |
| Contusion of upper limb (disorder) |
| Contusion of buttock (disorder) |
| Contusion of finger (disorder) |
| Contusion of foot (disorder) |
| Contusion of thumb (disorder) |
| Contusion of scapular region (disorder) |
| Contusion of thigh (disorder) |
| Contusion of upper arm (disorder) |
| Contusion of heel (disorder) |
| Contusion of lower limb (disorder) |
| Contusion of elbow (disorder) |
| Contusion with intact skin (disorder) |
| Contusion of forehead (disorder) |
| Contusion of multiple sites (disorder) |
| Contusion of multiple sites of lower limb (disorder) |
| Contusion of face (disorder) |
| Bruising symptom (finding) |
| Spontaneous bruising (disorder) |
| On examination - petechiae present (finding) |
| On examination - bruising (disorder) |
| Contusion, throat (disorder) |
| Contusion of anterior abdominal wall (disorder) |
| Contusion of coccyx (disorder) |
| Contusion, shoulder or upper arm (disorder) |
| Contusion of clavicular area (disorder) |
| Contusion, shoulder and upper arm, multiple sites (disorder) |
| Contusion, elbow or forearm (disorder) |
| Contusion wrist or hand (disorder) |
| Contusion, hand, excluding finger (disorder) |
| Contusion hand, palm (disorder) |
| Contusion hand, dorsum (disorder) |
| Contusion wrist, volar (disorder) |
| Contusion wrist, dorsum (disorder) |
| Contusion, fingernail (includes subungual hematoma) (disorder) |
| Contusion of multiple fingers (disorder) |
| Contusion, hip and thigh (disorder) |
| Contusion, knee and lower leg (disorder) |
| Contusion, ankle and foot, excluding toe(s) (disorder) |
| Contusion of eyelid (disorder) |
| Bruise of head (disorder) |
| Contusion of gingivae (disorder) |
| Contusion of face, scalp and neck, excluding eye(s) (disorder) |
| Superficial bruising (disorder) |
| Petechiae (disorder) |
| Superficial bruising of head and neck (disorder) |
| Contusion of pelvic region (disorder) |
| Contusion of lower back (disorder) |
| Contusion of sacral region (disorder) |
| Contusion of eye (disorder) |
| Hematoma (disorder) |
| Contusion of eye AND ocular adnexa (disorder) |
| Contusion of ocular adnexa and periocular tissues (disorder) |
| Easy bruising (finding) |
| Contusion, eye NOS (disorder) |
| Contusion, upper limb NOS (disorder) |
| Black eye NOS (disorder) |
| Contusion,trunk NOS (disorder) |
| Other contusion neck (disorder) |
| Contusion of face, scalp and neck NOS (disorder) |
| Contusion, lower limb and other unspecified sites (disorder) |
| Contusion, lower limb NOS (disorder) |
| Contusion, site NOS (disorder) |
| Bruising (finding) |
| Purpura (finding) |

| **Anaemia/Pallor (SNOMED Fully Specified Name)** |
| --- |
| On examination - color pale (finding) |
| On examination - anemia (finding) |
| On examination - equivocally anemic (disorder) |
| On examination - clinically anemic (disorder) |
| On examination - profoundly anemic (disorder) |
| Pale - symptom (finding) |
| Pale color saturation (qualifier value) |
| [D]Classification derived symptoms, signs and ill-defined conditions (situation) |
| [D]Symptoms affecting skin and other integumentary tissue (situation) |
| Pallor/perspiration (finding) |

| **Head/neck swelling/lump (SNOMED Fully Specified Name)** |
| --- |
| Swelling of structure of eye (finding) |
| Periorbital edema (disorder) |
| On examination - parotid swelling (finding) |
| On examination - submandibular swelling (finding) |
| On examination - sublingual swelling (finding) |
| On examination - unilateral thyroid swelling (situation) |
| On examination - thyroid swelling -bilateral (finding) |
| On examination - lip swelling (finding) |
| Swelling of eyelid (finding) |
| Swelling around eyes (finding) |
| Localized swelling, mass and lump, neck (finding) |
| Mass of head and/or neck (finding) |
| Head and neck swelling (finding) |
| Facial swelling (finding) |
| Mass of neck (finding) |
| Mass of ear structure (finding) |
| Pharyngeal swelling (finding) |
| Mass of head (finding) |
| Swelling of lower jaw region (finding) |
| Mass of nose (finding) |
| Mass of submental region (finding) |
| Mass of tongue (finding) |
| [D]Swelling, mass or lump in head or neck NOS (situation) |
| [D]Lump in head or neck (situation) |

MSK

| **Limb pain (SNOMED Fully Specified Name)** |
| --- |
| Pain in lower limb (finding) |
| Pain in finger (finding) |
| Foot pain (finding) |
| Hand pain (finding) |
| Thigh pain (finding) |
| Pain in limb (finding) |
| Growing pains (finding) |
| Pain in upper limb (finding) |
| Aching leg syndrome (finding) |
| Pain in left lower limb (finding) |
| Pain in right lower limb (finding) |
| Pain in limb - multiple (finding) |
| Pain in calf (finding) |
| Pain in forearm (finding) |
| Painful left arm (finding) |
| Pain in upper arm (finding) |

| **Joint pain (SNOMED Fully Specified Name)** |
| --- |
| Knee pain (finding) |
| Shoulder pain (finding) |
| Pain in wrist (finding) |
| Pain in elbow (finding) |

**Supplementary Table 6.** Number of cases with symptoms and their median diagnostic interval per cancer subtype. IQR=Inter-quartile range

| Cancer Subtype | No of cases (n, %) | No of cases with ≥1 symptom (n, %) | Median diagnostic interval (IQR) |
| --- | --- | --- | --- |
| Leukaemias |  |  |  |
|  | 769 | 398 (51.7) | 31 (4, 100) |
|  | 567 (73.7) | 313 (55.2) | 30 (5, 105) |
|  | 202 (26.3) | 85 (42.1) | 34 (4, 95) |
| Lymphomas |  |  |  |
|  | 672 | 394 (58.6) | 44 (18, 111) |
|  | 212 (31.5) | 121 (57.1) | 42 (15, 98) |
|  | 460 (68.5) | 273 (59.3) | 45 (22, 120) |
| CNS Tumours |  |  |  |
|  | 617 | 305 (49.4) | 26 (10, 76) |
|  | 404 (65.5) | 215 (53.2) | 21 (8, 70) |
|  | 213 (34.5) | 90 (42.2) | 37 (17, 79) |
| Sarcomas |  |  |  |
|  | 434 | 196 (45.2) | 60 (21, 113) |
|  | 207 (47.7) | 92 (44.4) | 44 (14, 102) |
|  | 227 (52.3) | 104 (45.8) | 70 (42, 115) |
| Abdominal Tumours |  |  |  |
|  | 263 | 151 (57.4) | 27 (7, 66) |
| Gonadal Germ Cell Tumours |  |  |  |
|  | 431 | 139 (32.0) | 28 (5, 89) |

**Supplementary Figure 1**


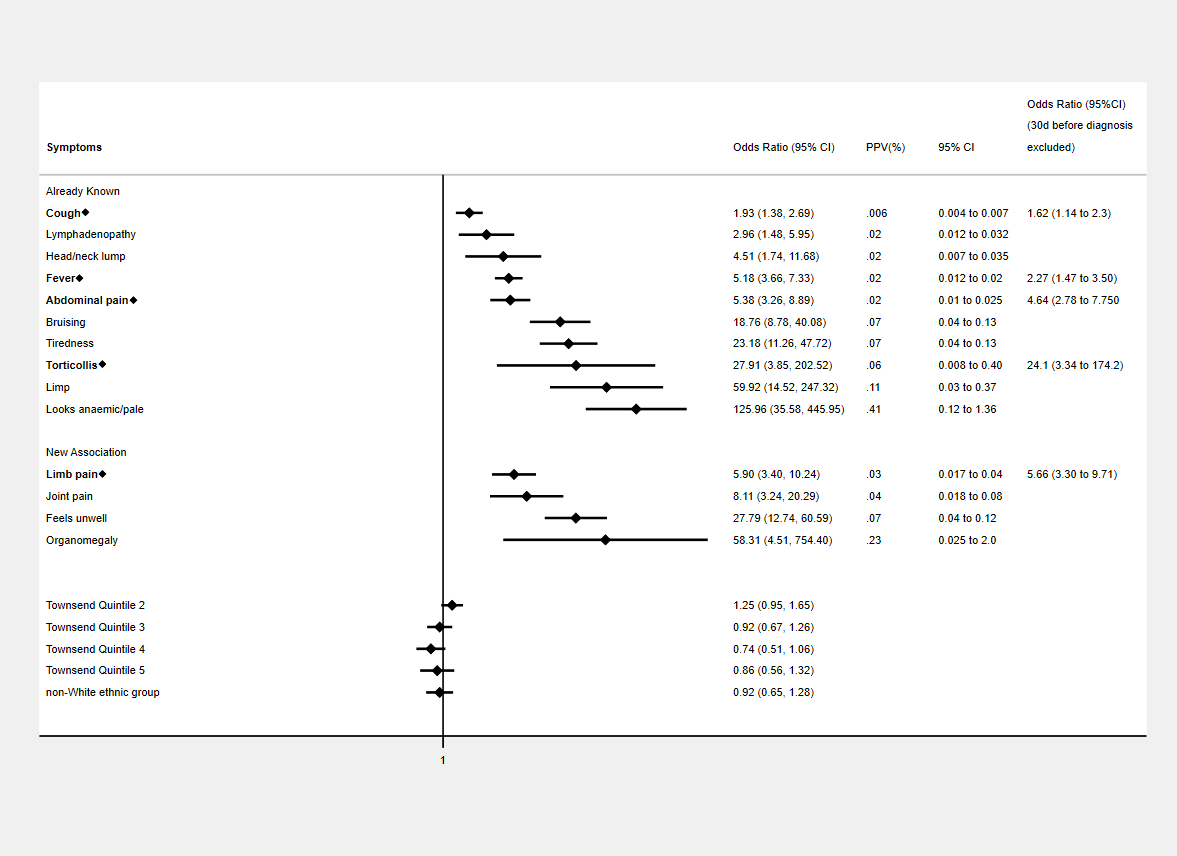

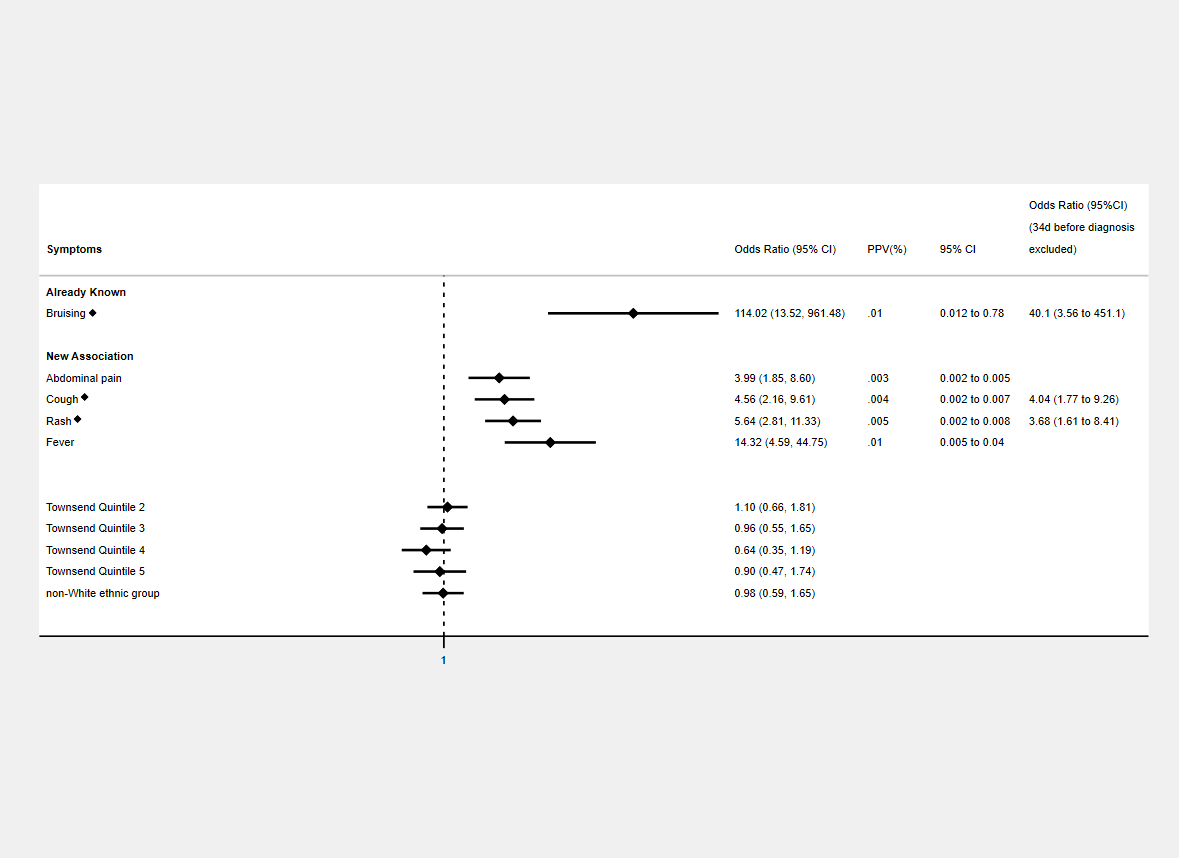


**Supplementary Figure 2**


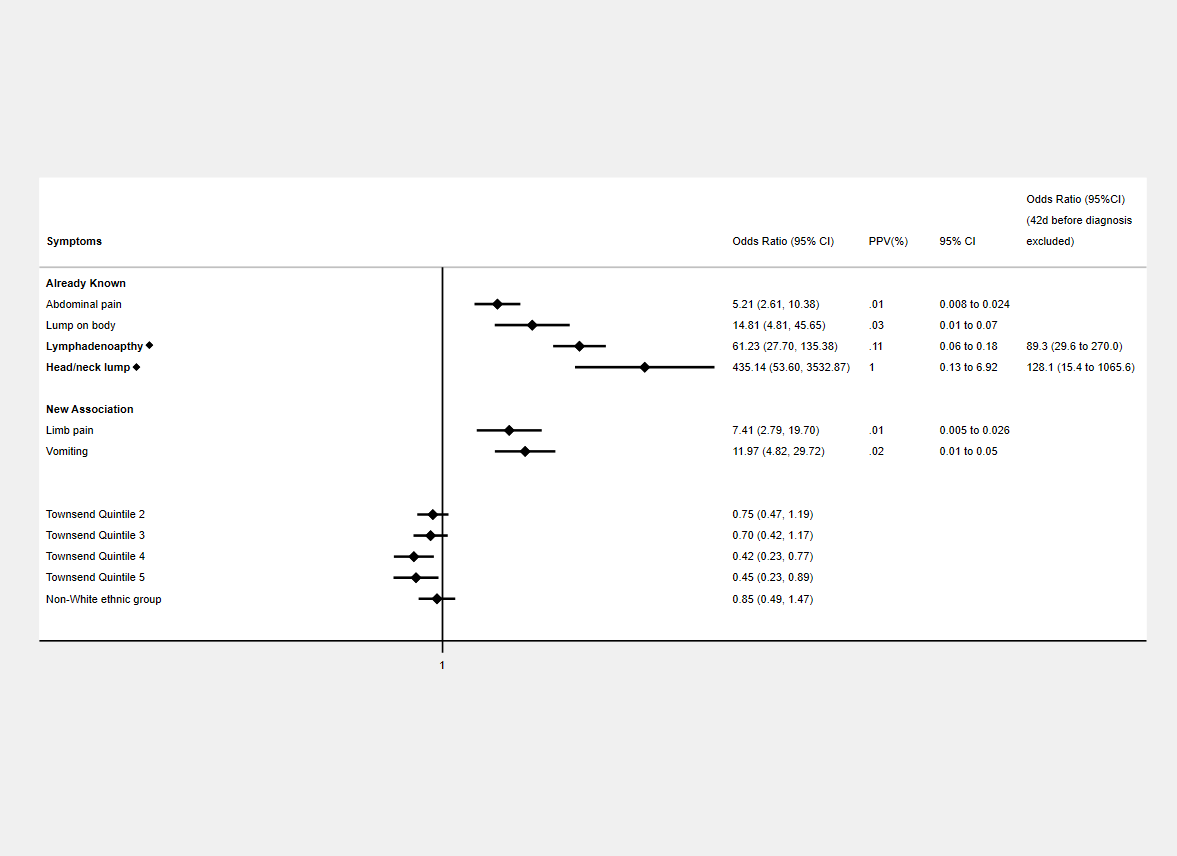

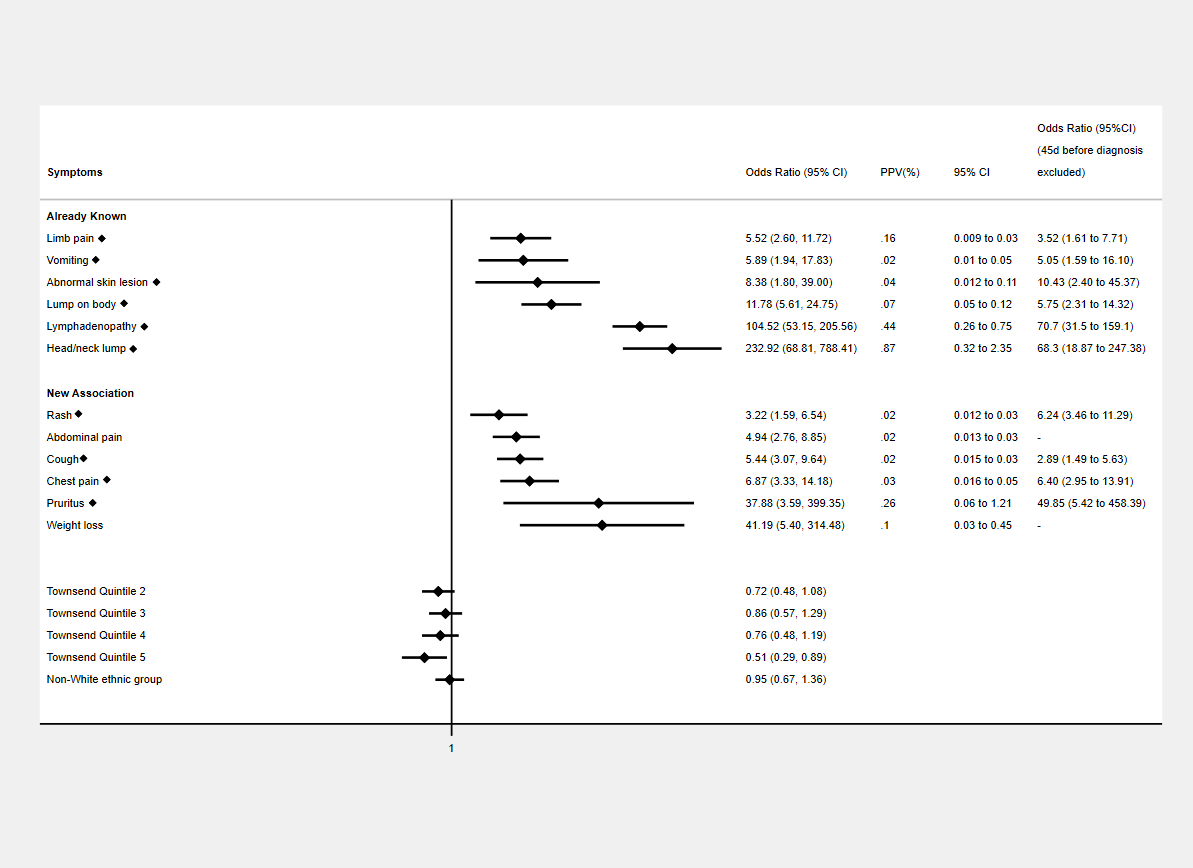


**Supplementary Figure 3**


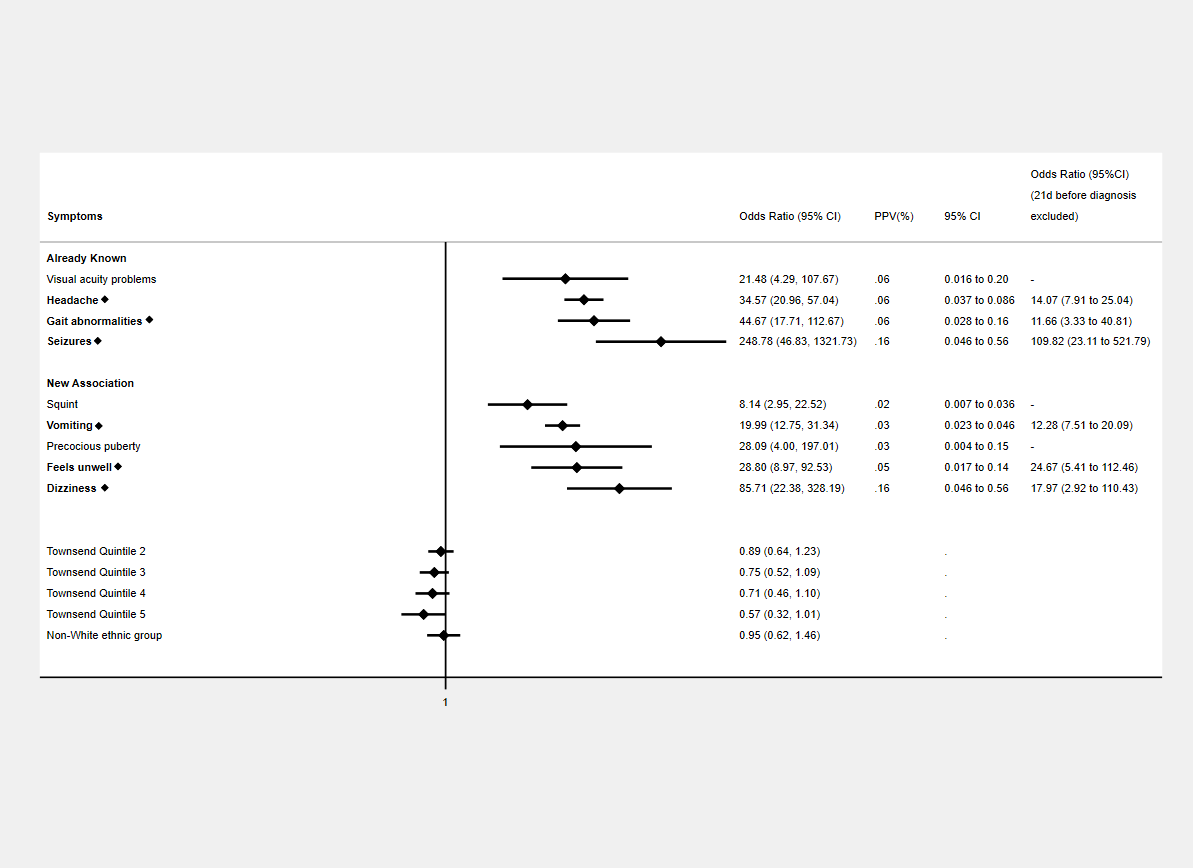

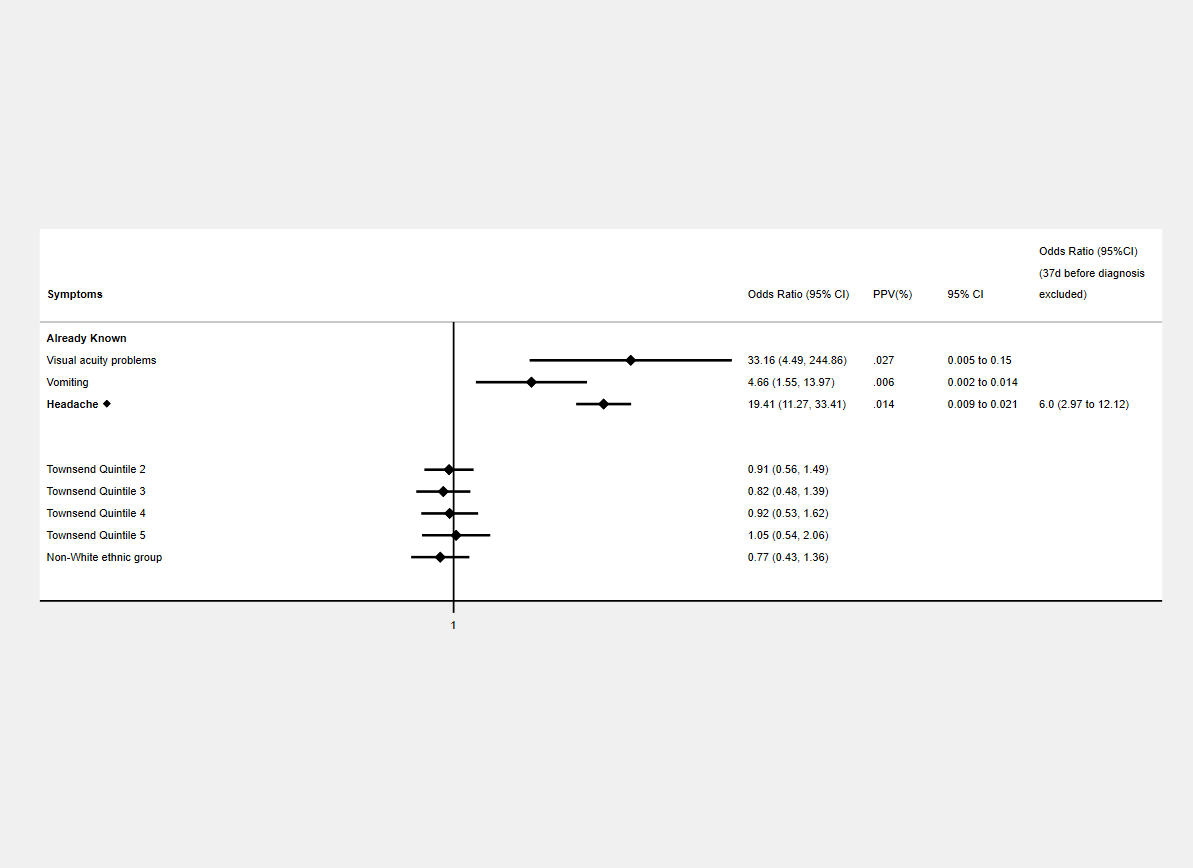


**Supplementary Figure 4**


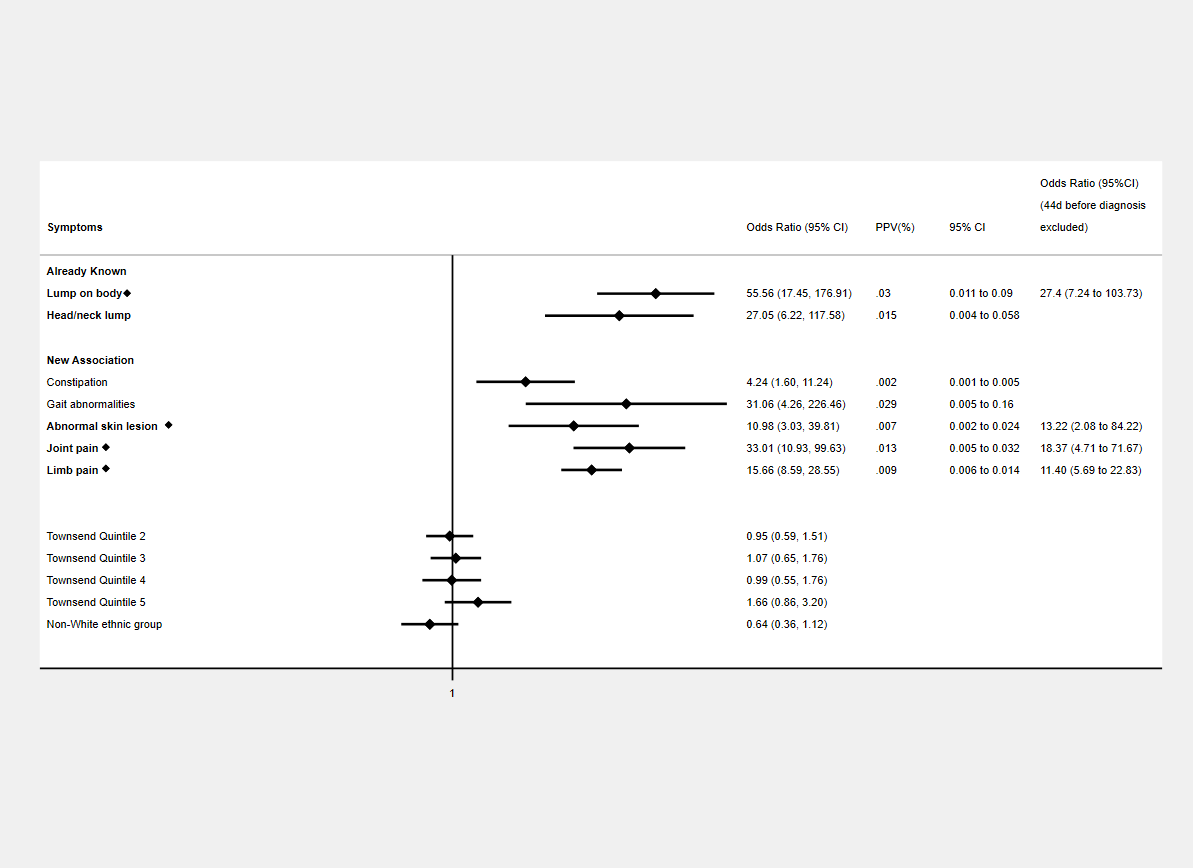

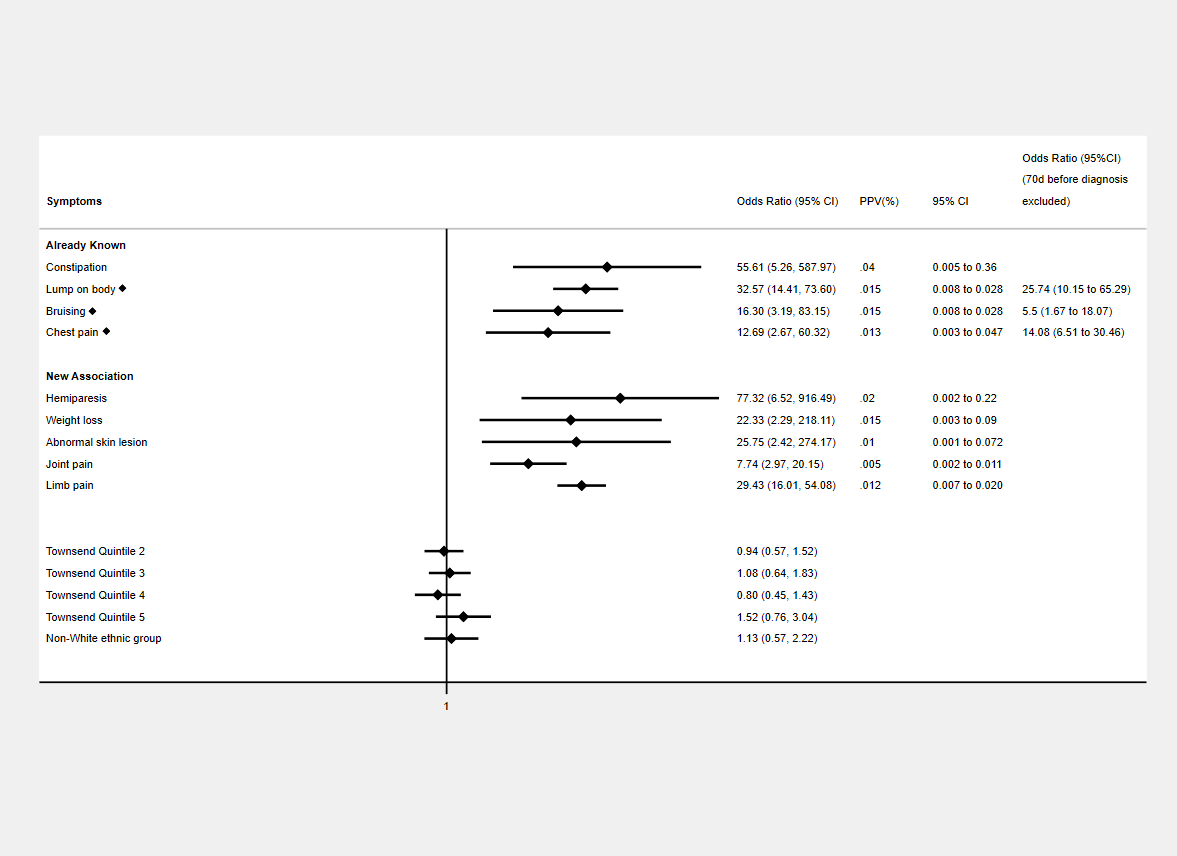

Supplement: Supplementary file 1 — Supplementary Appendix [file 41416_2024_2786_MOESM1_ESM.docx]
